# Multi-Omics Reveals the Impact of Exogenous Short-Chain Fatty Acid Infusion on Rumen Homeostasis: Insights into Crosstalk between the Microbiome and the Epithelium in a Goat Model

**DOI:** 10.1128/spectrum.05343-22

**Published:** 2023-07-13

**Authors:** Yongkang Zhen, Zanna Xi, Shaima Mohamed Nasr, Feiyang He, Mengli Han, Junliang Yin, Ling Ge, Yifei Chen, Yusu Wang, Wenjun Wei, Yihui Zhang, Mengzhi Wang

**Affiliations:** a College of Animal Science and Technology, Yangzhou University, Yangzhou, Jiangsu, People’s Republic of China; b State Key Laboratory of Sheep Genetic Improvement and Healthy Production, Xinjiang Academy of Agricultural Reclamation Sciences, Shihezi, Xinjiang, People’s Republic of China; c Experimental Farm of Yangzhou University, Yangzhou University, Yangzhou, Jiangsu, People’s Republic of China; Jilin University

**Keywords:** metabolism, microbiome, multi-omics, rumen homeostasis, ruminants, short-chain fatty acids

## Abstract

Emerging data have underscored the significance of exogenous supplementation of butyrate in the regulation of rumen development and homeostasis. However, the effects of other short-chain fatty acids (SCFAs), such as acetate or propionate, has received comparatively less attention, and the consequences of extensive exogenous SCFA infusion remain largely unknown. In our study, we conducted a comprehensive investigation by infusion of three SCFAs to examine their respective roles in regulating the rumen microbiome, metabolism, and epithelium homeostasis. Data demonstrated that the infusion of sodium acetate (SA) increased rumen index while also promoting SCFA production and absorption through the upregulation of SCFA synthetic enzymes and the mRNA expression of *SLC9A1* gene. Moreover, both SA and sodium propionate infusion resulted in an enhanced total antioxidant capacity, an increased concentration of occludin, and higher abundances of specific rumen bacteria, such as “*Candidatus* Saccharimonas,” *Christensenellaceae* R-7, *Butyrivibrio*, *Rikenellaceae* RC9 gut, and *Alloprevotella*. In addition, sodium butyrate (SB) infusion exhibited positive effects by increasing the width of rumen papilla and the thickness of the stratum basale. SB infusion further enhanced antioxidant capacity and barrier function facilitated by cross talk with *Monoglobus* and *Incertae Sedis*. Furthermore, metabolome and transcriptome data revealed distinct metabolic patterns in rumen contents and epithelium, with a particular impact on amino acid and fatty acid metabolism processes. In conclusion, our data provided novel insights into the regulator effects of extensive infusion of the three major SCFAs on rumen fermentation patterns, antioxidant capacity, rumen barrier function, and rumen papilla development, all achieved without inducing rumen epithelial inflammation.

**IMPORTANCE** The consequences of massive exogenous supplementation of SCFAs on rumen microbial fermentation and rumen epithelium health remain an area that requires further exploration. In our study, we sought to investigate the specific impact of administering high doses of exogenous acetate, propionate, and butyrate on rumen homeostasis, with a particular focus on understanding the interaction between the rumen microbiome and epithelium. Importantly, our findings indicated that the massive infusion of these SCFAs did not induce rumen inflammation. Instead, we observed enhancements in antioxidant capacity, strengthening of rumen barrier function, and promotion of rumen papilla development, which were facilitated through interactions with specific rumen bacteria. By addressing existing knowledge gaps and offering critical insights into the regulation of rumen health through SCFA supplementation, our study holds significant implications for enhancing the well-being and productivity of ruminant animals.

## INTRODUCTION

The increasing demand for sustainable and healthy production of domestic animal products, such as meat and milk, has become a pressing concern (1, 2). Ruminants, such as cattle, buffalo, sheep, and goats, play a pivotal role in meeting the global protein and energy consumption demands, providing 16% of global protein and 8% of global energy through milk and meat products (3). Therefore, improving the health of ruminants is crucial for enhancing global production in this sector.

The rumen, as a unique digestive and metabolic organ in ruminants, is the source of nutrition in digestive tract, and its homeostasis status plays a critical role in determining the efficient, healthy, and green production of ruminants (4). Failure to maintain proper rumen homeostasis can result in various nutritional metabolic diseases. Research indicates that an imbalance in rumen homeostasis can lead to the excessive release of toxic substances, including bacterial toxins or histamine (5). These substances subsequently stimulate the rumen epithelium, causing an increase in epithelial permeability, disruption of tight junctions, reduced self-repair capability, and compromised barrier functions (6). Eventually, these harmful substances will translocate into the blood circulation and trigger systemic pathological reactions (7). For example, the excess intake of a high-grain diet within a short period will induce subacute rumen acidosis (SARA) in goats (8), and SARA will damage the rumen epithelial barrier and ultimately leads to the inflammatory response and disease, such as diarrhea, liver abscess, and hoof diseases. SARA can even induce death in ruminants (9).

The rumen hosts a wide spectrum of microorganisms that play a leading role in fermenting plant fibers into short-chain fatty acids (SCFAs), which are absorbed across the rumen epithelium and used by the ruminants for maintenance and growth (10), where they provided more than 70% of the energy sources in ruminants (11). Evidence showed that SCFAs are not only used for energy supplements but also play an important role in regulating rumen development and the maintenance of health and homeostasis in ruminants (12). Previous reports also demonstrated that exogenous SCFA supplementation, particularly butyrate, not only enhances ruminal fermentation, maintains rumen microbiome colonization, and stimulates the development of rumen papillae and epithelial cells but also exhibits anti-inflammatory, antitumorigenic, and antimicrobial effects (13, 14). However, the role of other types of SCFA supplementation, such as with acetate and propionate, in regulating rumen development and maintaining homeostasis is less reported, while most of the studies were focus on SCFA absorption and metabolism and on rumen microbial fermentation (15, 16). Moreover, the impacts of high-concentration exogenous SCFA infusion on rumen homeostasis status, as well as the cross talk between rumen microbiome and rumen epithelium, are still unknown.

In short, it is important to study the effects of high-concentration exogenous SCFA infusion on rumen microbiome profiling and microbial metabolism patterns, as well as on rumen epithelial functions and homeostasis status. Thus, we hypothesized that massive infusion of exogenous SCFAs can regulate rumen microorganisms and their metabolic patterns, as well as rumen epithelial homeostasis, but without disturbing the normal function of the rumen. To address this hypothesis, we combined for the first time the infusion treatment of three SCFAs—acetate, propionate, and butyrate—to study their different roles using a *in vivo* goat model by multi-omics approaches. On the basis of numerous reports demonstrating that dietary SCFA supplementation can promote rumen development and homeostasis, therefore, the significance of our study is to evaluate the effects of high-concentration SCFA infusion on rumen fermentation patterns and epithelial homeostasis status in order to promote the healthy production of ruminants by precise nutrition regulation of SCFAs.

## RESULTS

### Rumen weight, rumen index, and rumen fermentation parameters in rumen contents.

**FIG 1:**
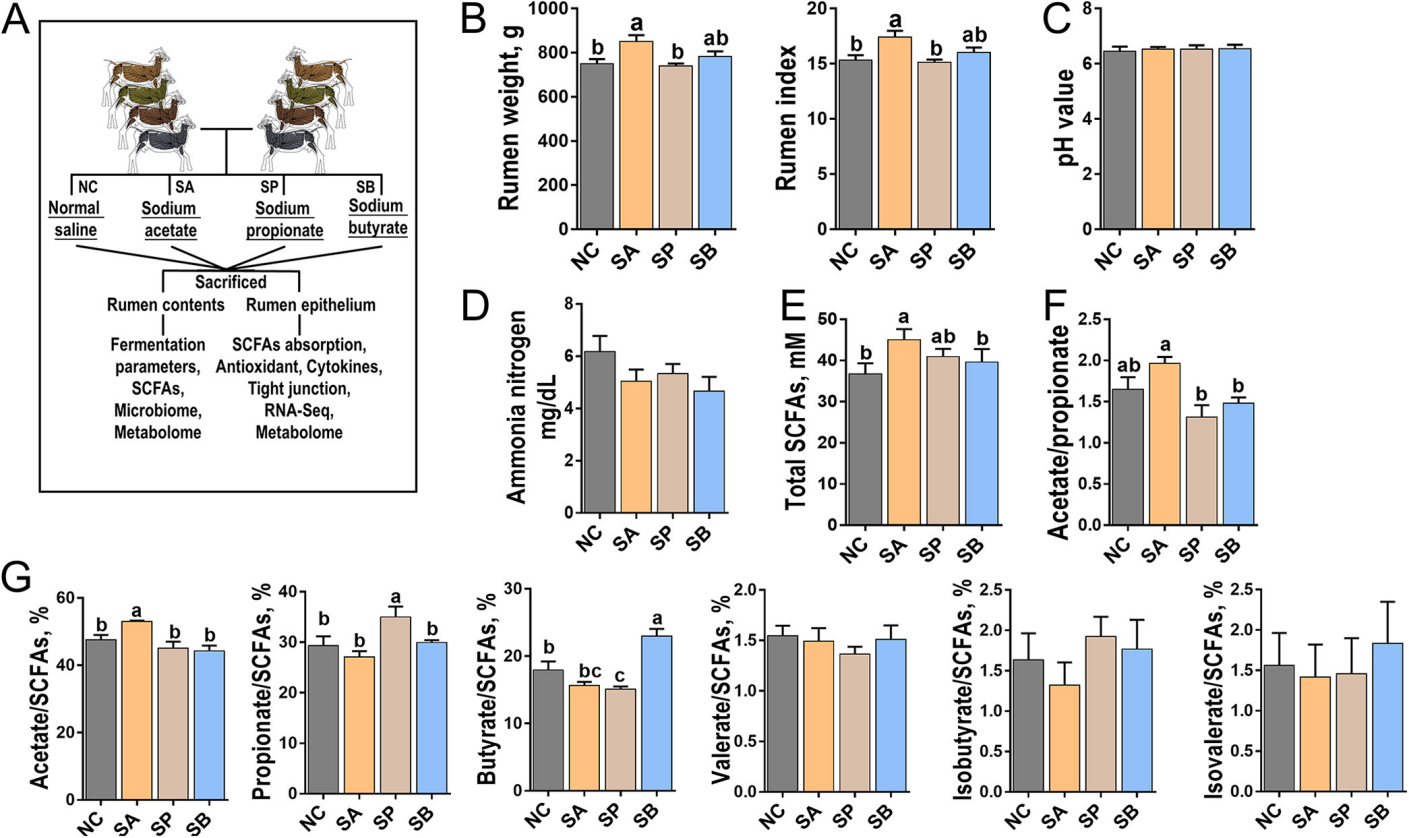
Schematic representation of experimental design and rumen parameters. (A) Schematic representation of experimental design. Goats were assigned to four groups to receive different infusion treatments of normal control using saline (NC), sodium acetate (SA), sodium propionate (SP), and sodium butyrate (SB) (*n* = 6/group). Representative charts of rumen weight and rumen index (B), pH value of rumen fluid (C), concentration of ammonia nitrogen in rumen fluid (D), concentration of total SCFAs in rumen fluid (E) were prepared. The acetate/propionate ratios in rumen fluid (F) and the proportions of acetate, propionate, butyrate, valerate, isobutyrate, and isovalerate in rumen fluid (G) were measured in four groups. Mean values with different letters are significantly different (*P < *0.05) according to Duncan’s multiple-range test. Data are shown as means ± the SD.

Figure 1 presents the rumen weights, rumen indices, ruminal pH values, ammonia nitrogen concentrations, and SCFA concentrations in the rumen contents for four groups. The sodium acetate (SA) group significantly increased rumen weight and rumen index (*P < *0.05) compared to the normal control using saline (NC) and sodium propionate (SP) groups (Fig. 1B). Meanwhile, the infusion of different SCFAs did not impact pH value or ammonia nitrogen concentration in rumen contents compared to the NC group (*P > *0.05) (Fig. 1C and D). In terms of SCFA concentration, the SA group significantly increased the concentration of total SCFAs compared to the NC and sodium butyrate (SB) groups and also increased the ratio of acetate to propionate compared to the SP and SB groups (Fig. 1E and F) (*P < *0.05). The infusion of SA significantly increased the proportion of acetate in rumen contents compared to the other groups (*P < *0.05), and a similar trend was found in the infusion of propionate or butyrate groups (*P < *0.05) (Fig. 1G). However, the infusion of three SCFAs did not impact the proportions of valerate, isobutyrate, and isovalerate compared to the NC group (*P > *0.05) (Fig. 1G).

### Relative abundance of SCFA synthetic enzymes in rumen contents and expression levels of SCFA transporter genes in rumen epithelial tissue.

**FIG 2:**
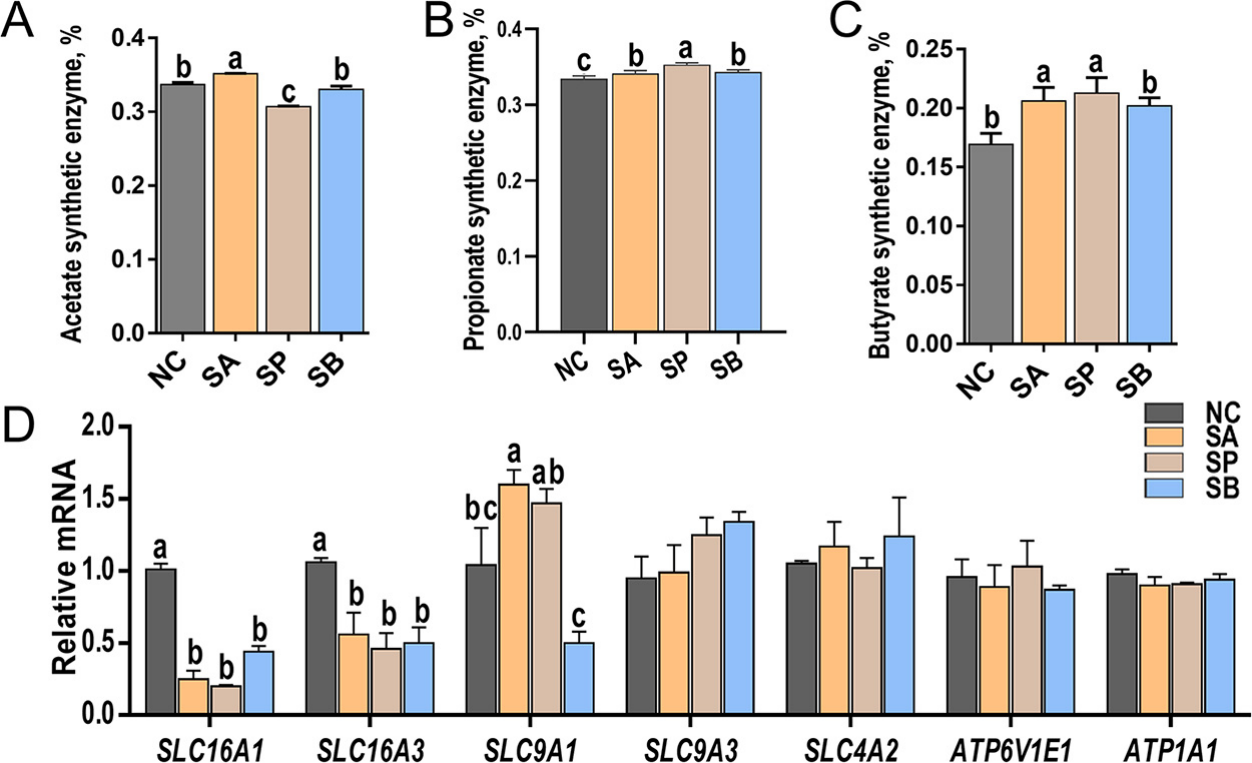
Relative abundances of SCFA synthetic enzymes and relative expression levels of SCFA transporter genes. Representative charts show the relative abundances of acetate synthetic enzymes (EC 6.3.4.3, EC 1.5.1.5, and EC 1.5.1.20) (A), propionate synthetic enzymes (EC 5.4.99.25, EC 5.1.99.1, and EC 6.2.1.1) (B), and butyrate synthetic enzymes (EC 2.3.1.9, EC 1.1.1.157, and EC 4.2.1.17) (C) of four groups in rumen fluid. Data were calculated based on the PICRUSt2 analysis of 16S rRNA sequencing, and the relative abundances of SCFA synthetic enzymes were the sum of the abundances of the genes that encoded the key enzymes. (D) Relative expression levels of *SLC16A1*, *SLC16A3*, *SLC9A1*, *SLC9A3*, *SLC4A2*, *ATP6V1E1*, and *ATP1A1* genes of rumen epithelial tissue determined using RT-PCR. Mean values with different letters are significantly different (*P < *0.05) according to Duncan’s multiple-range test. Data are shown as means ± the SD (*n* = 6/group).

The relative abundance of SCFA synthetic enzymes in rumen contents was calculated using a PICRUSt2 approach with 16S rRNA sequencing data. The data indicated significant differences in the relative abundance of synthetic enzymes due to SCFA infusion (Fig. 2A to C). Specifically, the SA group significantly increased the relative abundance of the acetate synthetic enzyme, while the SP group significantly reduced its relative abundance compared to the NC group (*P < *0.05) (Fig. 2A). Moreover, the SP group had the highest abundance of the propionate synthetic enzyme, followed by the SA and SB groups, while the NC group had the lowest abundance (*P < *0.05) (Fig. 2B). Finally, the infusion of all three SCFAs significantly increased the relative abundance of the butyrate synthetic enzyme in rumen contents compared to the NC group (*P < *0.05) (Fig. 2C).

Subsequently, the relative expression levels of SCFA transporter genes in rumen epithelial tissue were detected using reverse transcription-PCR (RT-PCR). The data indicated significant alterations in the expression of SCFA transporters genes due to the different SCFA infusions (Fig. 2D). Specifically, the relative expression levels of *SLC16A1* and *SLC16A3* genes were significantly downregulated in all three SCFA infusion groups compared to the NC group (*P < *0.05). As for the *SLC9A1* gene, the SA and SP groups significantly upregulated its expression level, while the SB group downregulated its expression level compared to the NC group (*P < *0.05). However, the expression levels of other SCFA transporter genes, such as *SLC9A3*, *SLC4A2*, *ATP6V1E1*, and *ATP1A1*, were not significantly different among the four groups (*P > *0.05).

### Structure, morphology, and homeostasis parameters of rumen epithelial tissue.

**FIG 3:**
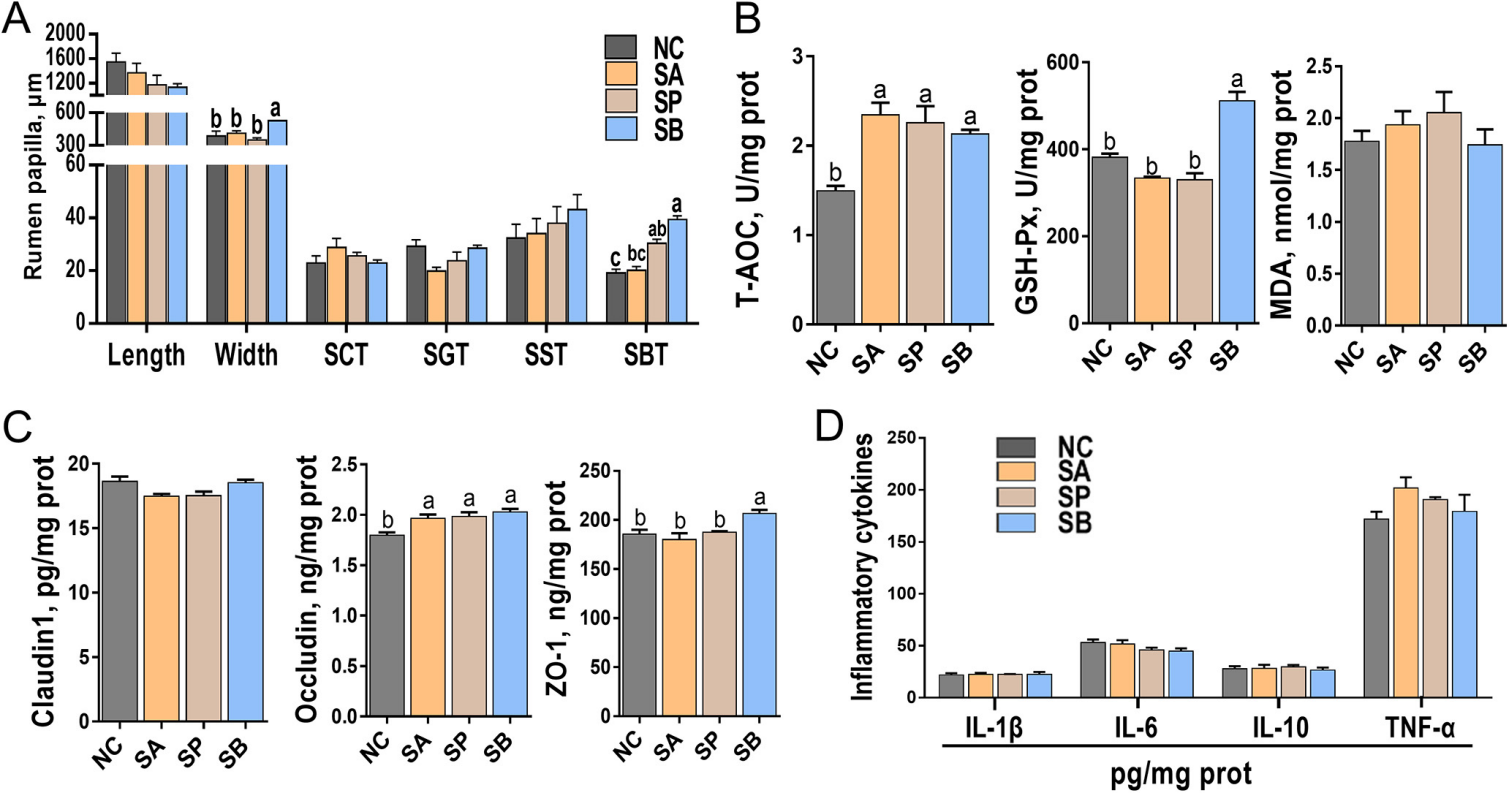
Development of rumen papilla, antioxidant capacity parameters, and concentrations of tight junctions and inflammatory cytokines of rumen epithelial tissue. (A) Representative charts of the length and width of rumen papilla, SCT, SGT, SST, and SBT. (B) Representative charts of T-AOC, the activity of GSH-Px, and the content of MDA of rumen epithelium. (C) Representative charts of the activities of Claudin1, occludin, and ZO-1 of rumen epithelium. (D) Representative charts of the activities of IL-1β, IL-6, IL-10, and TNF-α of rumen epithelium. Mean values with different letters are significantly different (*P < *0.05) according to Duncan’s multiple-range test. Data are shown as means ± the SD (*n* = 6/group).

Figure 3 (see also Fig. S1 in the supplemental material) illustrates the structure, morphology, antioxidant capacity, tight-junction concentrations, and inflammatory cytokine concentrations in rumen epithelial tissue. The representative light micrographs of rumen papillae cross sections in Fig. S1 demonstrated intact morphology and structure of rumen papillae in both the SCFA infusion and NC groups. There were no differences observed in the length of rumen papilla among the four groups (*P > *0.05). However, the SB group exhibited a significant increase in the width of rumen papilla compared to the other groups (*P < *0.05) (Fig. 3A). In addition, the SP and SB groups displayed an increased stratum basale thickness (SBT) compared to the NC group (*P < *0.05). However, the infusion of the three SCFAs did not affect the stratum corneum thickness (SCT), the stratum granulosum thickness (SGT), and the stratum spinosum thickness (SST) compared to the NC group (*P > *0.05) (Fig. 3A).

Furthermore, the total antioxidant capacity (T-AOC) and the enzymatic activity of glutathione peroxidase (GSH-Px), as well as the malondialdehyde (MDA) content in rumen epithelial tissue, were examined (Fig. 3B). The data indicated that the infusion of the three SCFAs significantly increased T-AOC compared to the NC group (*P < *0.05). Regarding GSH-Px, the SB group showed a significant increase in its activity, while the SA and SP groups exhibited no significant changes compared to the NC group (*P < *0.05). Furthermore, the MDA content did not differ significantly among the groups (*P > *0.05).

Moreover, the protein concentrations of tight junctions and inflammatory cytokines in rumen epithelial tissue were measured using an enzyme-linked immunosorbent assay (ELISA), with their standard curves presented in Fig. S2. All standard curves displayed an *R*^2^ value greater than 0.99, confirming the reliability of the data. Figure 3C demonstrates the significant impact of the three SCFAs on the protein concentrations of tight junctions. For instance, compared to the NC group, the concentration of occludin was significantly increased in all three SCFA infusion groups (*P < *0.05). In addition, the SB group displayed an increased concentration of tight-junction protein 1 (ZO-1) compared to the NC group (*P < *0.05). However, the infusion of the three SCFAs did not affect the concentration of Claudin1 compared to the NC group (*P > *0.05). Finally, the protein concentrations of inflammatory cytokines, including interleukin-1β (IL-1β), IL-6, IL-10, and tumor necrosis factor alpha (TNF-α), were not significantly affected by the infusion of the three SCFAs compared to the NC group (*P > *0.05).

### Microbiota structure and diversity in rumen contents.

**FIG 4:**
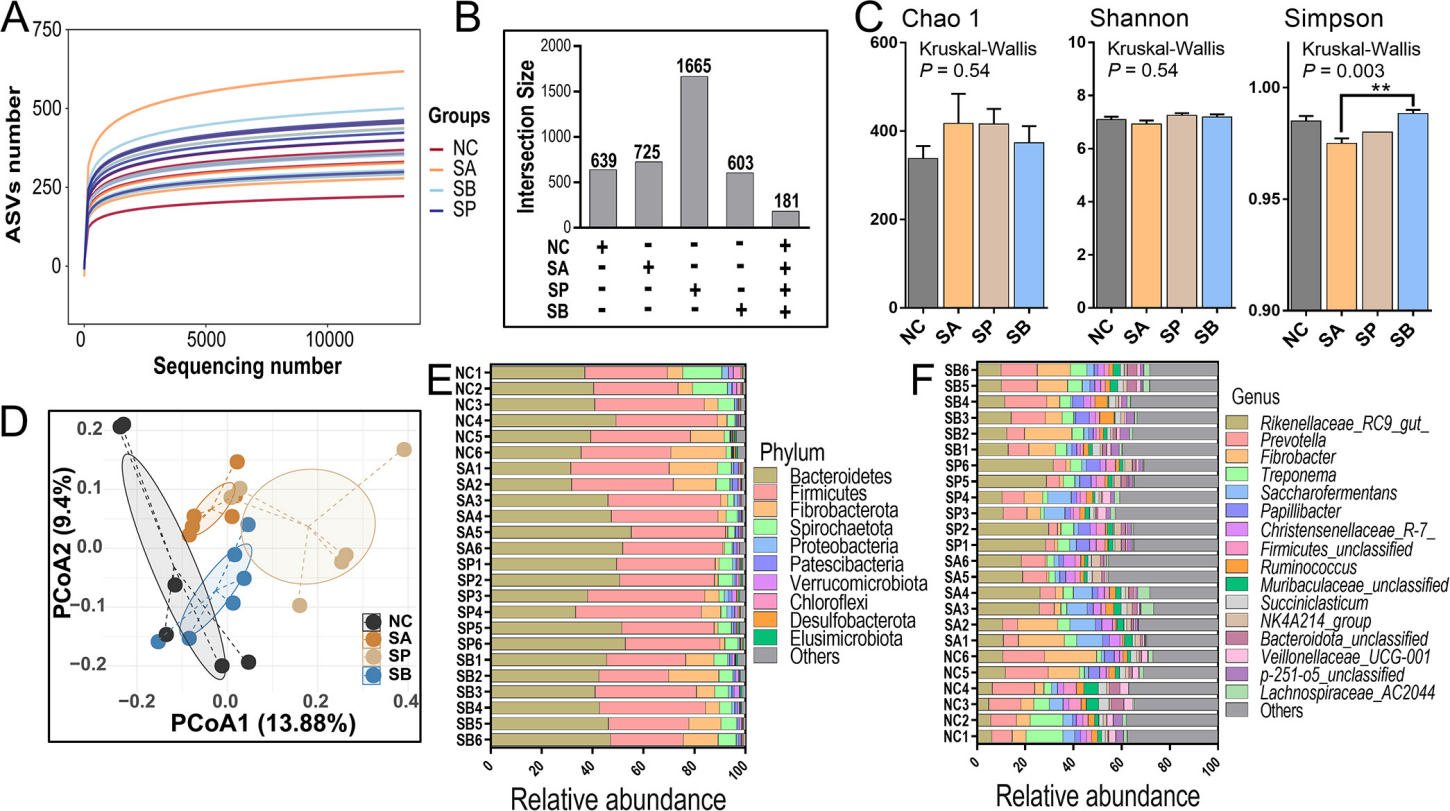
Rumen bacterial diversities and compositions. (A) Diagram of the rarefaction curve of rumen bacteria. (B) UpSet diagram of the numbers of unique or shared ASVs in rumen fluid of four groups. (C) Representative charts of microbial α-diversity indexes of Chao1, Shannon, and Simpson indexes. Data are shown as means ± the SD, while statistical analyses were conducted using Kruskal-Wallis tests (**, *P < *0.01). (D) PCoA of microbial β-diversity calculated using unweighted UniFrac distances. (E and F) Component proportions of rumen bacteria in phylum and genus levels.

Figure 4 and Fig. S3 underscore the alterations in the diversity and taxonomic differences of rumen microbiome in three SCFA infusion and NC groups. First, the rarefaction curve of the amplicon sequence variant (ASV) number indicated that a rising trend of the horizontal coordinate sequence number of the dilution curve of the four groups was flat after 10,000, and the slope of the curve was smooth with small changes, indicating that the sequencing volume of each group had been sufficient (Fig. 4A). Then, the number of observed ASVs based on 16S rRNA sequencing ranged from 334 to 370, and the sequencing coverage exceeded 99% of each group (see Fig. S3A and B), and no difference was observed among four groups (*P > *0.05). Then, the UpSet diagram revealed that numbers of unique bacterial ASVs in rumen contents for the NC, SA, SP, and SB groups were 639, 725, 1,665, and 603, respectively, and the number of shared ASVs was 181 (Fig. 4B). Meanwhile, the microbial α-diversity indicated that infusion of three SCFAs had no impact on the Chao1 and Shannon indexes compared to the NC group (*P > *0.05); however, SB increased the Simpson index compared to the SA group (*P < *0.05) (Fig. 4C). The rarefaction curves of microbial Chao1, Shannon, and Simpson indexes also indicated the reliability of our data (see Fig. S3C to E). Meanwhile, the β-diversity calculated using principal coordinate analysis (PCoA) diagram based on the unweighted UniFrac distance also revealed that the microbial structure among four groups were slightly separated (Fig. 4D). Moreover, data also demonstrated that different treatments had different bacterial relative abundances at the phylum and genus levels. For example, rumen bacteria with higher abundances mainly included *Bacteroidetes*, *Firmicutes*, *Fibrobacterota*, *Spirochaetota*, and *Proteobacteria* at the phylum level (Fig. 4E) and *Rikenellaceae* RC9 gut, *Prevotella*, *Fibrobacter*, Treponema, *Saccharofermentans*, *Papillibacter*, *Christensenellaceae* R-7, and *Ruminococcus* at the genus level (Fig. 4F). The relative abundances of *Bacteroidetes* and *Firmicutes*, and the ratio of *Firmicutes* to *Bacteroidetes*, were not different among the four groups (see Fig. S3F).

**FIG 5:**
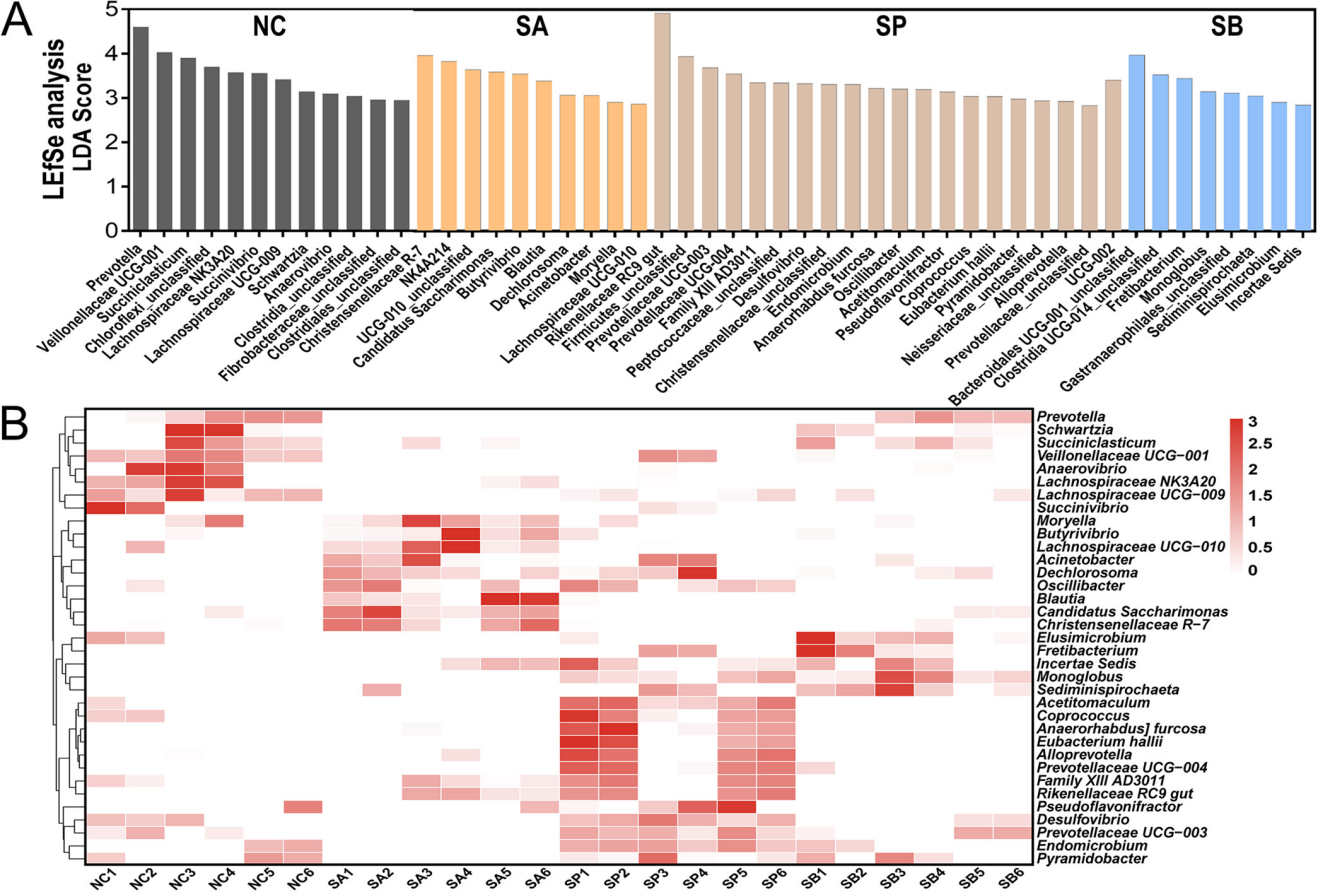
Selection of candidate rumen bacteria in response to SCFA infusion. (A) LEfSe analysis of candidate rumen bacteria and different microbial taxonomy in response to SCFA infusion at the genus level. Only LDA scores of >2.5 are marked. (B) Cluster heatmap of differential rumen bacteria.

Significant taxonomic differences in bacterial genus classification among the four groups were further analyzed using linear discriminant analysis effect size (LEfSe) (Fig. 5A). LEfSe results were visualized using a taxonomy bar chart, and only linear discriminant analysis (LDA) scores of >2.5 were marked in our study. The results indicated that, at the bacterial genus level, *Veillonellaceae* UCG-001, *Schwartzia*, *Succiniclasticum*, *Succinivibrio*, *Prevotella*, *Lachnospiraceae* NK3A20 group, *Anaerovibrio*, and *Lachnospiraceae* UCG-009 were enriched in NC (Fig. 5A); *Moryella*, *Lachnospiraceae* UCG-010, “*Candidatus* Saccharimonas,” *Blautia*, *Dechlorosoma*, NK4A214 group, *Christensenellaceae* R-7 group, Acinetobacter, and *Butyrivibrio* were enriched in SA; *Rikenellaceae* RC9 gut, *Prevotellaceae* UCG-003, *Prevotellaceae* UCG-004, *Family XIII AD3011*, *Desulfovibrio*, *Endomicrobium*, Anaerorhabdus furcosa, *Oscillibacter*, *Acetitomaculum*, *Pseudoflavonifractor*, *Coprococcus*, Eubacterium hallii, *Pyramidobacter*, *Alloprevotella*, and UCG-002 were enriched in SP; and *Fretibacterium*, *Monoglobus*, *Sediminispirochaeta*, *Elusimicrobium*, and *Incertae Sedis* were enriched in SB. The cluster results shown in Fig. 5B also revealed that the relative abundances of significant taxonomic differences in the bacterial genus of each group were higher than those of other groups.

### Metabolome profile and functional enrichment in rumen contents.

**FIG 6:**
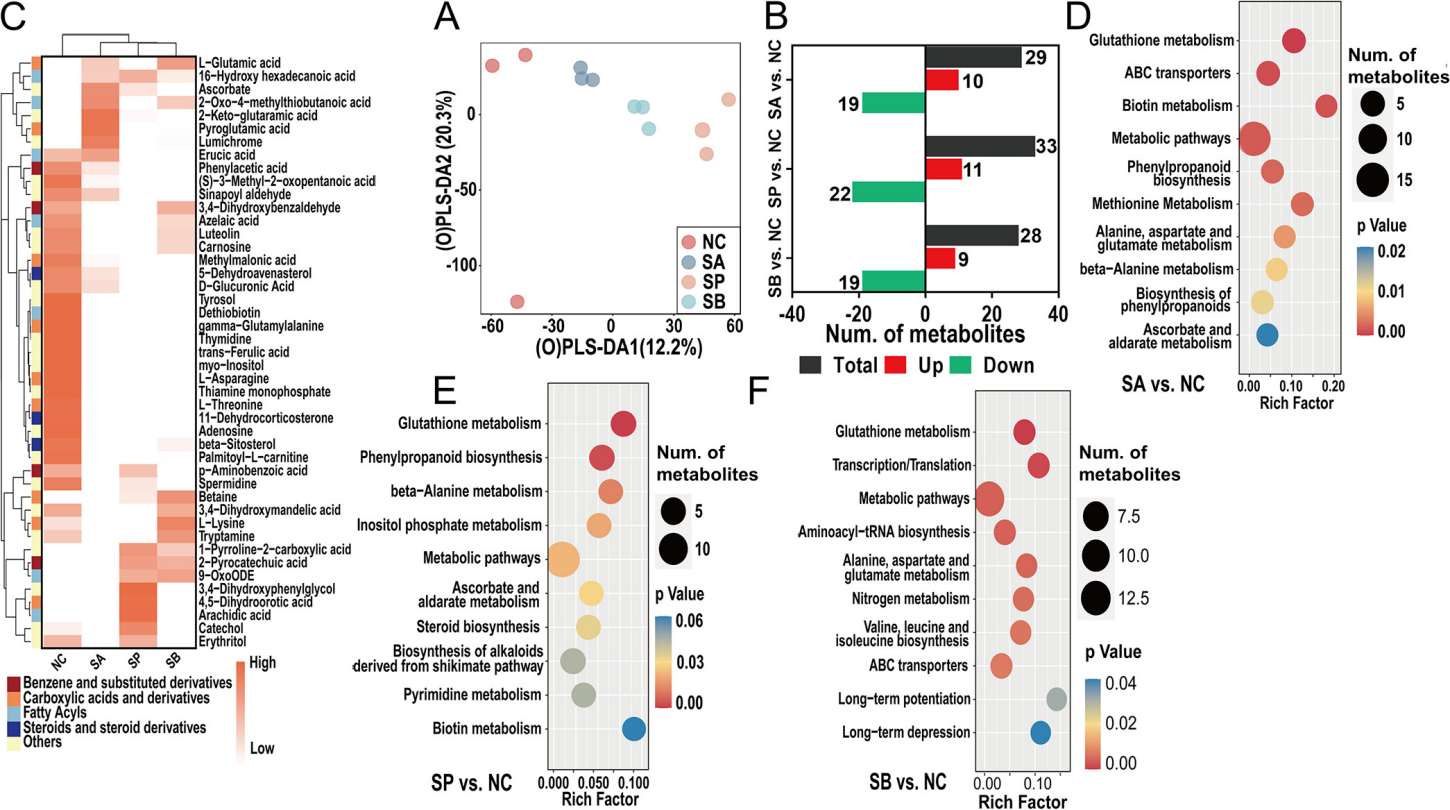
Metabolome profiles and the functional enrichment analysis of rumen fluid. (A) Distributions of LC-MS metabolome data using an OPLS-DA score plot. (B) Number of differential metabolites of three comparisons. (C) Cluster heatmap of the differential metabolites and the classification information. (D to F) Functional enrichment analysis of differential metabolites using KEGG database of three comparisons.

The rumen contents contained not only unabsorbable foods but also a mixture of metabolites produced by rumen bacteria; therefore, it is necessary to detect the metabolome profile in rumen contents. Multivariate statistical methods were applied to analyze metabolome data based on liquid chromatography-mass spectrometry (LC-MS). First, 386 small molecules were accurately identified among the four groups (see Fig. S4A), and a principal-component analysis (PCA) was performed on all samples to monitor the robustness of sample preparation and the stability of instrument analysis, and the data indicated that PCA was reliable for profiling metabolic variations (see Fig. S4B). The orthogonal partial least squares discrimination analysis (OPLS-DA) model was then used to aid in the selection of differential metabolites among the four groups, and a 200× permutation test was also performed to test the reliability of the current model. The data clarified that the metabolic pattern in rumen contents of the four groups was significantly separated on the OPLS-DA axis and the OPLS-DA model was reliable for distinct metabolite screening (Fig. 6A; see also Fig. S4C).

Metabolites with a variable importance in projection (VIP) threshold of >1 in the OPLS-DA model and univariate statistical significance (*P < *0.05) were selected as differential metabolites, and their numbers among three comparisons are shown in Fig. 6B. A total of 29 metabolites were significantly altered between the SA and NC groups; among them, 10 metabolites increased (such as ascorbate, sodium deoxycholate, tryptophanol, and pantothenic acid), while 19 metabolites decreased (such as adenosine, l-asparagine, l-lysine, betaine, and carnosine) (see Table S1). Meanwhile, the number of differential metabolites between SP and NC groups was 33, which 11 were increased, such as catechol, sodium deoxycholate, arachidic acid, and tryptophanol, whereas 22 metabolites were reduced, such as UMP, adenosine, azelaic acid, carnosine, methylmalonic acid, and luteolin (see Table S1). Finally, 9 metabolites were significantly increased (such as l-glutamic acid, tryptophanol, 2-pyrocatechuic acid, and threonic acid) and 19 metabolites were significantly decreased (such as erucic acid, thymine, phenylacetic acid, catechol, and l-threonine) between the SB and NC groups (see Table S1). A cluster heatmap was used to identify the differentially altered metabolites and obtain their classification information (Fig. 6C).

Finally, a functional enrichment analysis of differential metabolites using the KEGG database and a correlation analysis between differential metabolites and rumen bacteria were performed to reveal the key microbial metabolic pathways that were affected by SCFA infusion. As shown in Fig. 6D, most metabolites were enriched in metabolic pathways, glutathione metabolism, ABC transporters, biotin metabolism, and methionine metabolism pathways between the SA and NC groups. Meanwhile, the infusion of sodium propionate altered the pathways such as glutathione metabolism, phenylpropanoid biosynthesis, and beta-alanine metabolism compared to the NC group (Fig. 6E). It is also worth noticing that SB also affected pathways such as metabolic pathways; glutathione metabolism; transcription/translation; alanine, aspartate, and glutamate metabolism; nitrogen metabolism; long-term potentiation; and long-term depression compared to the NC group (Fig. 6F). Lastly, Fig. S5 underscores that most metabolites were highly correlated with rumen microbes according to the correlation heatmap. For example, metabolites such as l-asparagine, luteolin, spermidine, adenosine, myo-inositol, azelaic acid, beta-sitosterol, and carnosine were highly negatively correlated with rumen microbes such as *Blautia*, Acinetobacter, *Rikenellaceae* RC9 gut, *Prevotellaceae* UCG-004, *Family XIII AD3011*, Anaerorhabdus furcosa, *Oscillibacter*, Eubacterium hallii, while metabolites such as catechol, arachidic acid, pyroglutamic acid, lumichrome were highly positively correlated with such rumen microbiomes (*P < *0.05, *P < *0.01, or *P < *0.001).

### Transcriptome profile and functional enrichment in rumen epithelial tissues.

**FIG 7:**
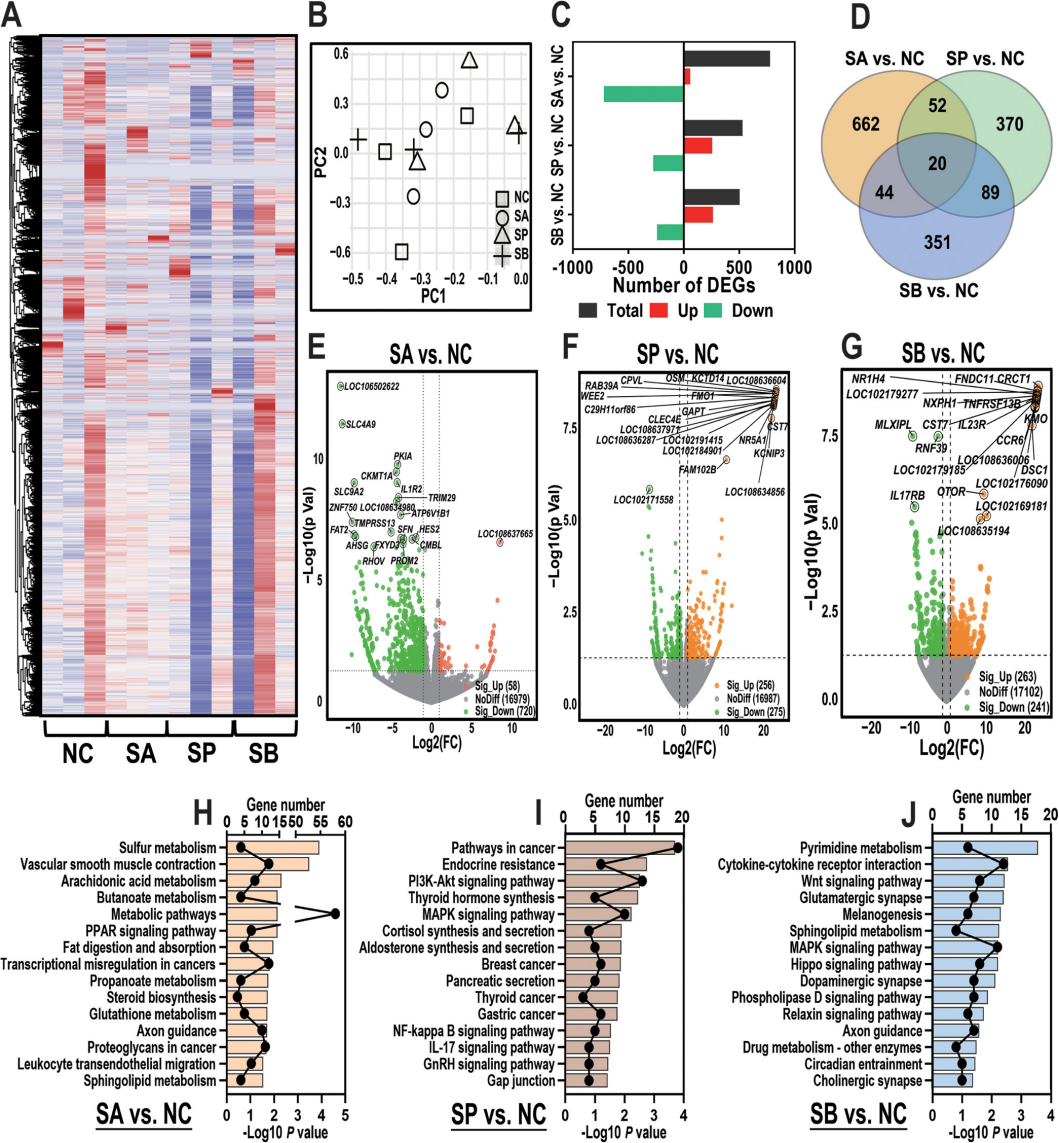
Transcriptome sequencing profiles and functional enrichment analysis of rumen epithelial tissue. (A) Heatmap of 25,220 genes that were accurately identified among four groups using transcriptome sequencing data. (B) PCA using a combined data set of all genes of four groups. (C) Number of DEGs of three comparisons. (D) Venn diagram of the number of singly expressed or coexpressed DEGs of three comparisons. (E to G) Volcano plot of the DEGs of three comparisons. Orange dots represent upregulated DEGs, and green dots represent downregulated DEGs. (H to J) Functional enrichment analysis of upregulated and downregulated DEGs using the KEGG database of three comparisons.

We predicted that the alterations in rumen microbiome and metabolome induced by infusion of three SCFAs could further dysregulate the gene expression level in rumen epithelial tissue. Therefore, an RNA-seq study was used to detect the gene expression level. First, a total of 25,220 mRNA was accurately identified among four groups after strictly quality control and clustering analysis (Fig. 7A). Then, PCA using a combined data set with gene counts of >0 indicated that the original RNA-seq data did not separate on both PC1 and PC2 among four groups (Fig. 7B).

Next, the expression levels of differentially expressed genes (DEGs) were further analyzed using a cutoff *P* value of <0.05, and the number of DEGs among the three comparisons is shown in Fig. 7C. As shown in Fig. 7E, between the SA and NC groups, 778 DEGs were screened; 58 DEGs were upregulated (such as *SIM2*, *FAM102B*, *MAP3K19*, *TP53TG5*, *MYOZ3*, and *FAM117A*), while 720 DEGs were downregulated (such as *SLC4A9*, *PKIA*, *IL1R2*, *SLC9A2*, *TRIM29*, and *ATP6V1B1*) (see Table S2). Meanwhile, 256 DEGs were significantly upregulated (such as *CLEC4E*, *GAPT*, *FMO1*, *CPVL*, *OSM*, *NR5A1*, and *FAM102B*), while 275 DEGs were significantly downregulated (such as *NEGR1*, *LAT*, *SATB2*, *TSKS*, *GPR162*, *CCR5*, and *BCAN*) between the SP and NC groups (Fig. 7F; see also Table S2). Finally, 504 DEGs were screened between the SB and NC groups. Among them, 263 DEGs were significantly upregulated (such as *IL-7*, *TNFSF13B*, *MUC21*, *FAM102B*, *CXCL13*, *IL23R*, and *NR1H4*), while 241 DEGs were significantly downregulated (such as *IL17RB*, *SLC7A14*, *CDCA8*, *WNT3*, *SLC13A5*, *SLC22A14*, and *SLC30A2*) (Fig. 7G; see also Table S2). Moreover, Fig. 7D demonstrates the number of DEGs expressed exclusively in each comparison and the 20 conserved DEGs that were ubiquitous in all comparisons (such as *ADAMTS14*, *EGR3*, *ARID5A*, *MME*, *FAM102B*, *CPVL*, *HS3ST4*, *PKHD1*, *RRAD*, and *LDLR*). A detailed list of singly expressed or coexpressed DEGs among the three comparisons is shown in Table S3.

Lastly, the upregulated and downregulated genes were analyzed using the KEGG database in order to assess their functional consequence. The enrichment analysis results indicated that infusion of three SCFAs regulated different functions and pathways; for example, the infusion of sodium acetate significantly altered the genes that were enriched in several metabolic pathways, such as sulfur metabolism, arachidonic acid metabolism, butanoate metabolism, the PPAR signaling pathway, fat digestion and absorption, and propanoate metabolism (Fig. 7H). Meanwhile, it is also worth noticing that the infusion of sodium propionate affected the pathways that were related to the inflammation process, such as endocrine resistance, the mitogen-activated protein kinase signaling pathway, gastric cancer, the NF-κB signaling pathway, and the IL-17 signaling pathway. Finally, DEGs that were involved in pathways such as pyrimidine metabolism, glutamatergic synapse, melanogenesis, dopaminergic synapse, axon guidance, circadian entrainment, and cholinergic synapse were related to sodium butyrate infusion treatment (Fig. 7J).

### Metabolome profile and functional enrichment in rumen epithelial tissue.

**FIG 8:**
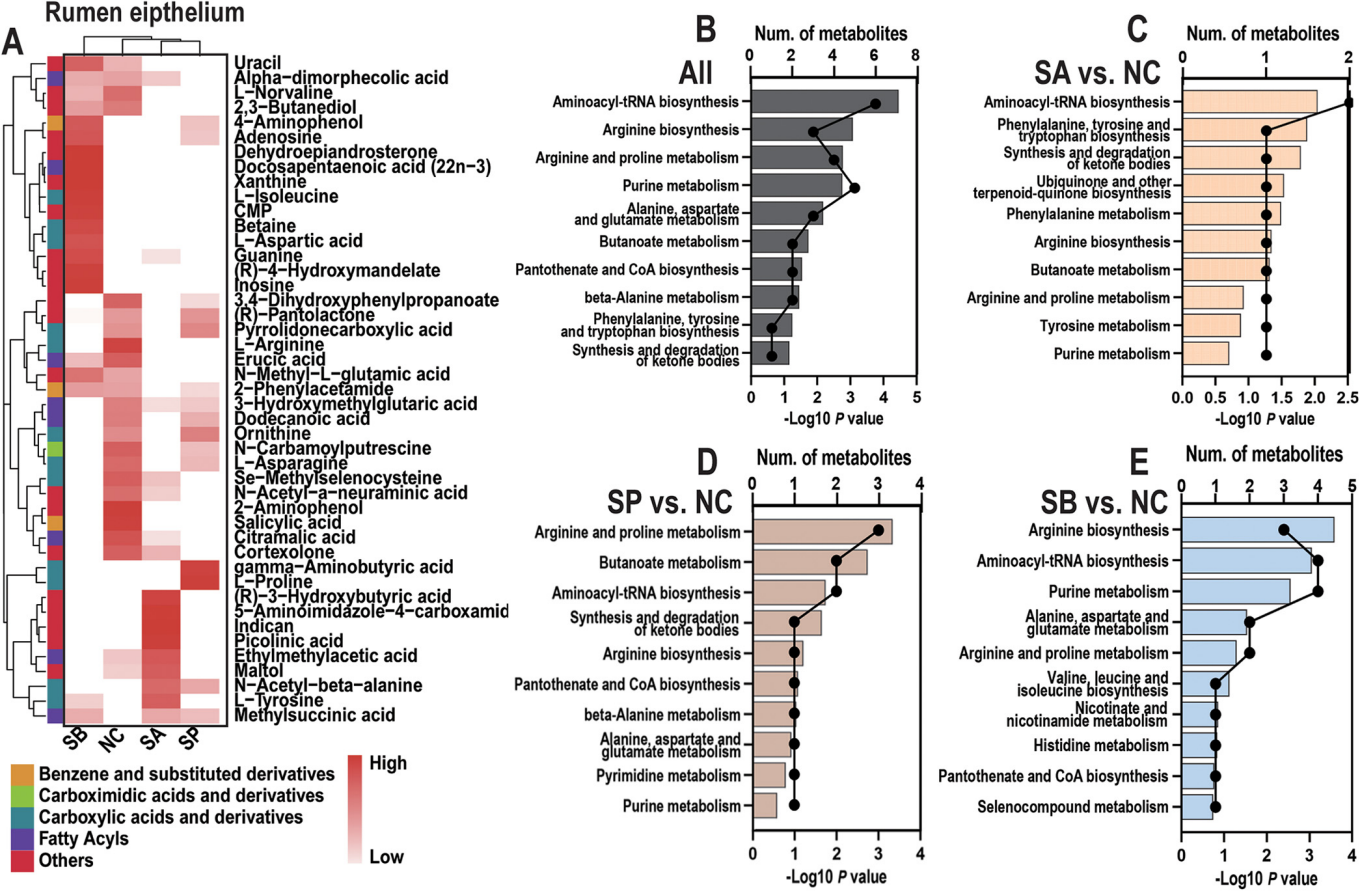
Metabolome profiles and functional enrichment analysis of rumen epithelial tissue. (A) Cluster heatmap of all differential metabolites and their classification information of rumen epithelial tissue. (B to E) Functional enrichment analysis of all differential metabolites and the specific differential metabolites of three comparisons determined using the KEGG database.

Finally, the LC-MS metabolome was accessed again in order to explore the metabolic profile in rumen epithelial tissue of different SCFA infusions. Less than rumen contents, a total of 326 small molecules was identified among four groups in rumen epithelial (see Fig. S5A). The PCA score chart, the OPLS-DA score chart, and the 200× permutation test also indicated that the metabolic pattern was different among the four groups in rumen epithelium (see Fig. S5B to D). In detail, 14 metabolites were significantly altered in the comparison between the SA and NC groups; 6 were increased [such as (*R*)-3-hydroxybutyric acid, l-tyrosine, and picolinic acid], while 8 were decreased (such as erucic acid, 2-phenylacetamide, l-arginine, and l-norvaline) (see Table S4). Meanwhile, 12 metabolites were increased (such as betaine, l-aspartic acid, CMP, guanine, l-isoleucine, adenosine, xanthine, and inosine), while 11 metabolites were decreased (such as cortexolone, l-asparagine, ornithine, and l-arginine) between the SP and NC groups (see Table S4). Finally, five metabolites (such as gamma-aminobutyric acid, adenosine, l-proline, and methylsuccinic acid) were increased, while 14 metabolites were decreased (such as cortexolone, l-norvaline, erucic acid, uracil, maltol, and l-arginine) between the SB and NC groups (see Table S4). A merged list of these differential metabolites is presented in Fig. 8A.

The KEGG enrichment results of differential metabolites are shown in Fig. 8B to E; it is interesting that the oral infusion of three SCFAs significantly affected amino acid and fatty acid biosynthesis and metabolism processes (Fig. 8B). For example, infusion of sodium acetate affected pathways such as aminoacyl-tRNA biosynthesis; phenylalanine, tyrosine, and tryptophan biosynthesis; and phenylalanine metabolism (Fig. 8C). Meanwhile, pathways like arginine and proline metabolism, butanoate metabolism, and aminoacyl-tRNA biosynthesis were enriched selected between the SP and NC groups (Fig. 8D), and arginine biosynthesis, aminoacyl-tRNA biosynthesis, and purine metabolism pathways were significantly altered between the SB and NC groups (Fig. 8E).

### Correlation analysis between rumen epithelial homeostasis parameters and multi-omics data.

**FIG 9:**
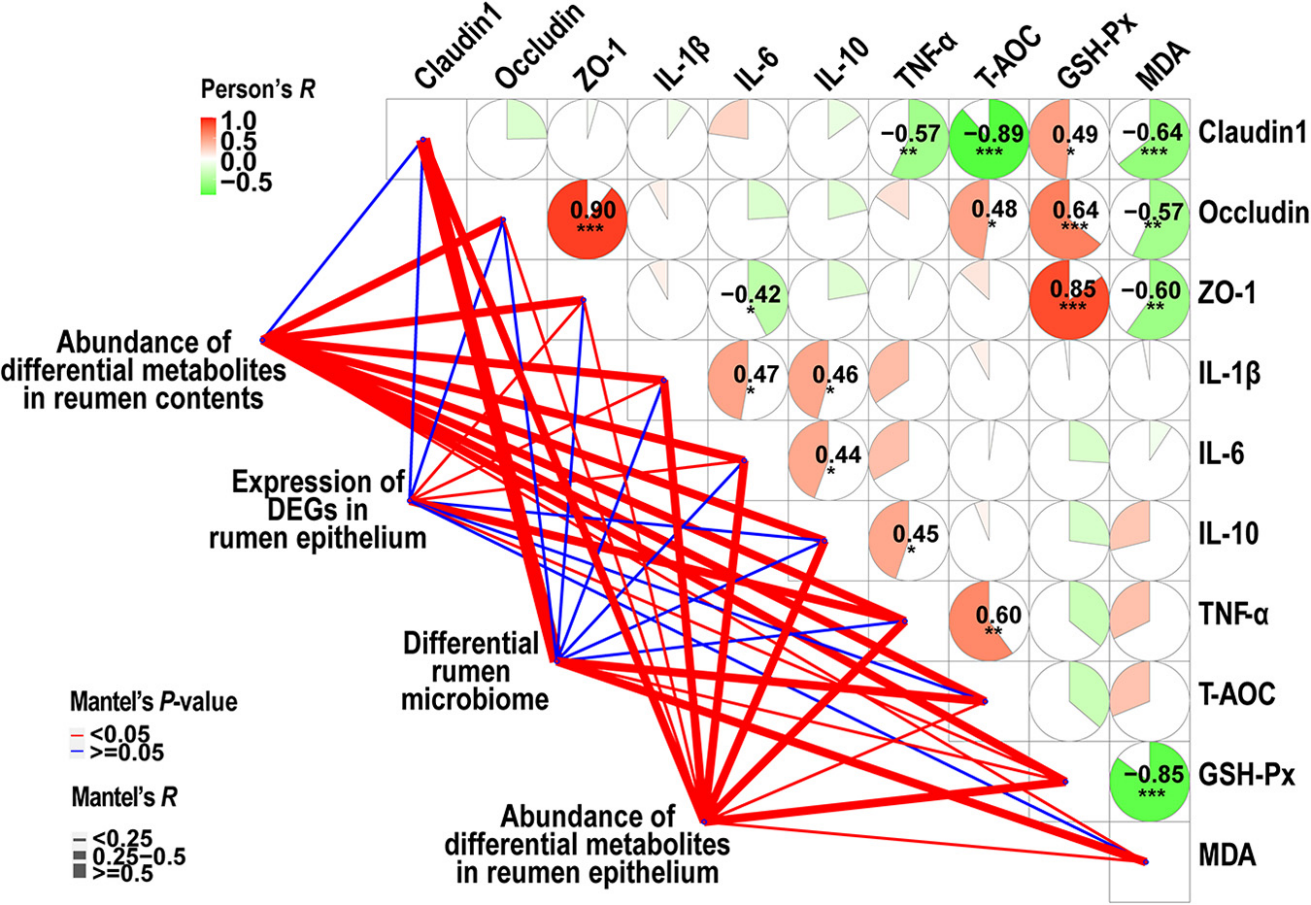
Correlation analysis of rumen homeostasis parameters and multi-omics data. Correlation analyses of rumen homeostasis parameters—such as antioxidant capacity parameters, tight junctions, and inflammatory cytokines—were performed using Pearson’s correlation coefficient (*, *P < *0.05; **, *P < *0.01; ***, *P < *0.001). The correlation analysis among rumen homeostasis parameters and multi-omics data was performed using a Mantel test. A red line indicates a Mantel test *P* value of <0.05, and a blue line indicates a Mantel test *P* value of >0.05.

Results corroborated an interesting correlation among ruminal homeostasis parameters (such as antioxidant capacity, tight junctions, and inflammatory cytokines) and multi-omics data using both Pearson’s correlation and Mantel test analysis (Fig. 9). For example, for Pearson’s correlation analysis, the concentration of GSH-Px was highly positive correlated with ZO-1 (Pearson’s *R *= 0.85, *P < *0.001); meanwhile, the concentration of ZO-1 was also positively positive correlated with occludin (Pearson’s *R *= 0.90, *P < *0.001), and the content of MDA was highly negatively correlated with GSH-Px (Pearson’s *R* = −0.85, *P < *0.001). Moreover, Mantel’s test revealed that these homeostasis parameters were associated with abundance of differential metabolites in both rumen contents and epithelium, as well as the expression levels of DEGs in the rumen epithelium and differential rumen microbiome (Mantel’s *P < *0.05).

## DISCUSSION

In this investigation, we performed a goat model to assess the effects of massive SCFA infusion on various parameters related to rumen fermentation, rumen microbiome and derived metabolism, rumen epithelial homeostasis (including ruminal development, antioxidant capacity, tight junctions, and inflammatory cytokines), and ruminal transcriptome and metabolism profiles.

Crucial indicators of ruminal fermentation, such as pH value and ammonia nitrogen concentration, serve as vital reflections of microbial metabolism status. Our findings demonstrated that the pH value ranged between 6.31 and 6.53, while the ammonia nitrogen concentration in rumen contents ranged from 4.67 to 6.18 mg/dL across the four experimental groups. These levels were deemed suitable for the colonization, fermentation, and metabolism of ruminal microorganisms. Previously established reports have defined subacute ruminal acidosis (SARA) in goats when the ruminal pH remains below 5.5 for 3 h per day (17). However, our study observed no occurrence of SARA during the infusion of the three SCFAs. In terms of SCFAs, the infusion of sodium acetate significantly elevated its concentration. A similar trend was observed in the propionate and butyrate infusion groups. Typically, increased concentrations of total SCFAs led to a lower rumen pH (18). Nevertheless, no change in ruminal pH was observed despite the increased SCFA concentrations. Notably, the infusion of sodium acetate also raised the concentration of total SCFAs. This observation led us to speculate that the higher concentration of total SCFAs in the sodium acetate group may be attributed to an increased relative abundance of acetate, propionate, and butyrate synthetic enzymes in rumen contents. In addition, our data indicated that the infusion of the three SCFAs affected their absorption by decreasing the expression levels of major SCFA transporter genes, such as *SLC16A1* and *SLC16A3*, as well as enhancing the expression level of *SLC9A1* gene in the SA group. Previous studies have also reported that high concentrations of sodium butyrate can inhibit its own transport by downregulating the abundance of *SLC16A1* and *SLC16A3* in rumen epithelial cells *in vitro*, and our *in vivo* experiments with goats reaffirmed this result (19). Consequently, our data suggested that the infusion model was successfully established and could be employed for subsequent investigations.

Regarding rumen epithelial development and homeostasis parameters, our data provided novel insights into the substantial impact of massive infusion of the three major SCFAs on antioxidant capacity, reinforcement of rumen barrier function, and promotion of rumen papilla development without inducing rumen epithelial inflammation. Notably, infusion of sodium acetate and sodium butyrate demonstrated particularly consistent results with our hypothesis. Specifically, sodium butyrate infusion significantly increased the width of rumen papilla and the thickness of the stratum basale compared to the control group, thereby facilitating ruminal papilla growth. These observations aligned with previous reports indicating that enhanced rumen development contributes to molecular adaptations in nutrient absorption and metabolism within the rumen epithelium (20–22). Improved rumen development further enhanced antioxidant capacity and barrier function, as evidenced by increased total antioxidant capacity and the activity of GSH-Px, as well as the concentrations of occludin and ZO-1 proteins in the rumen epithelium compared to the control group. Consequently, our findings indicated that massive sodium butyrate supplementation can serve as a stimulator and antioxidant for ruminal epithelium growth and function, not only in young ruminants but also in gestating dairy goats (20, 23, 24). Furthermore, sodium butyrate has been identified as a positive regulator in cases of rumen homeostasis dysfunction. For instance, sodium butyrate can mitigate damage to rumen epithelial barrier function, local inflammation, and destruction induced by high-concentrate diets, thereby preserving epithelial integrity in goats (25, 26). Our data provided additional support for this notion, as evidenced by lower concentrations of inflammatory cytokines, such as IL-1β, IL-6, and TNF-α, in our treatment groups.

Numerous studies have confirmed the role of rumen microorganisms in maintaining and regulating the structural integrity and barrier function of the rumen epithelium through interactions with the host (27). For example, a previous study reported that early microbiome composition, including *Prevotella*, *Bacteroides*, and *Ruminococcus*, actively contributes to rumen development and barrier functions through its interaction with the host transcriptome in young ruminants (28). Another study revealed that the rumen microbiota-mediated gut-liver axis is crucial for liver inflammation and health, with *Ruminococcus*, *Solobacterium*, and *Syntrophococcus* in the rumen being potential microbial markers of liver disorders (29). Furthermore, ruminal *Olsenella*, *Methanosphaera*, *Quinella*, “*Candidatus* Saccharimonas,” and *Methanobrevibacter* have been reported to enhance immune status by increasing the concentration of serum IgG in goats (30). Moreover, significant shifts in the ruminal microbiome and metabolites associated with inflammation responses have been observed during disease conditions such as mastitis, indicating that rumen microorganisms have a significant impact not only on rumen epithelial inflammation but also on other peripheral tissues or organs (31). In our study, the pattern of rumen microbial fermentation was substantially altered by the infusion of the three SCFAs. Specifically, sodium acetate increased the relative abundance of “*Candidatus* Saccharimonas,” *Christensenellaceae* R-7 group, and *Butyrivibrio* compared to the other groups. These bacterial genera have been associated with beneficial health and digestive system functions in both humans and ruminants (32). For instance, a higher relative abundance of *Christensenellaceae* R-7 has been observed in stool samples from healthy individuals compared to those with inflammatory bowel disease (33). These results suggested that sodium acetate infusion may also confer health benefits on goats by increasing the rumen index, total antioxidant capacity, and occludin levels. In addition, we found that the bacterial genera enriched in the sodium butyrate group are associated with various metabolic processes. For example, *Monoglobus* has been shown to decompose and utilize pectins in the human colon (34), and the *Incertae Sedis* genus has been implicated in ammonia assimilation through the rumen epithelial wall (35). Together, these microorganisms may contribute to enhancing rumen homeostasis in the sodium butyrate infusion group. In summary, our findings supported the notion that the ruminal microbiome forms a closely connected symbiotic relationship with the ruminal epithelium during long-term evolution, and the homeostasis within the rumen microbial ecosystem is critical for healthy production (36).

Finally, we evaluated the metabolic patterns in both rumen contents and epithelium based on metabolome and RNA-seq data. Interestingly, the infusion of the three SCFAs led to distinct regulatory effects on ruminal metabolism. For instance, sodium acetate infusion primarily affected fatty acid and amino acid metabolism in both rumen contents and epithelium. Specifically, alterations were observed in glutathione metabolism and methionine metabolism in rumen contents, as well as phenylalanine metabolism, arginine and proline metabolism, butanoate metabolism, and tyrosine metabolism in rumen epithelium. RNA-seq data also indicated that the sodium acetate group affected pathways such as arachidonic acid metabolism, butanoate metabolism, propanoate metabolism, sphingolipid metabolism, and glutathione metabolism, as well as pathways related to lipid metabolism, including fat digestion and absorption and PPAR signaling pathways. These findings were consistent with previous studies that highlight the role of acetate as the main substrate for *de novo* synthesis of fatty acids in ruminants through acetyl-coenzyme A (37). Acetate provides most of the carbon and approximately half of the reducing equivalents (NADPH) required for *de novo* lipogenesis through the isocitrate pathway (isocitrate dehydrogenase), with the remaining NADPH derived from glucose metabolism through the pentose phosphate pathway (38). Our data further supported the notion that acetate availability is crucial for meeting energy requirements and milk fat synthesis in ruminants (39). Furthermore, our data strongly suggested that butyrate may act as a signaling molecule connecting rumen microbes to communicate with the host. For example, the metabolome data revealed that sodium butyrate altered signal transmission pathways such as long-term potentiation and long-term depression, as well as glutamatergic synapse, dopaminergic synapse, cholinergic synapse, and circadian entrainment, based on RNA-seq data. Numerous studies have demonstrated that butyrate can modulate the phosphorylation, acetylation, and methylation levels of intracellular proteins, thereby influencing the expression of related genes and cell signaling. The concentration of butyrate in circulation, tissues, and gut luminal contents are known to play a critical role in regulating metabolic disorders, inflammation, and lipid metabolism (40–42). Notably, we previously reported that circadian rhythm disorder induced by the knockout of the *Per2* gene increased intestinal butyrate concentration in mice, providing further evidence of the connection between butyrate and the host’s circadian rhythm, not only in mice but also in ruminants (43).

## MATERIALS AND METHODS

### Ethics approval statement.

All animal experiments were performed according to the ethical policies and procedures approved by the Animal Care and Use Committee of Yangzhou University, Jiangsu, China (approval 202203-512).

### Animals, diets, and experimental design.

A total of 24 healthy multiparous Guanzhong goats with an average body weight (BW) of 47.44 ± 3.38 kg at 1.5 years old in the early lactation period were selected in this study. All of the selected goats were maintained at the same farming condition and fed a standard diet containing 60% forage and 40% concentrate mix (dry matter basis) (see Table S5) *ad libitum* for 14 days of continuous stable feeding. The diet was formulated meeting the current feeding recommendations. All goats were housed individually in their tie stalls. They had free access to water and were fed two times a day at 8 a.m. and 18 p.m.

All goats were then randomly divided into four groups (*n* = 6 for each group) after adaption with the diet and light conditions to receive different SCFA infusion treatments. They were assigned to a normal control group infusion with normal saline (NC), an infusion with sodium acetate solution at 0.8 g/kg BW (SA), an infusion with sodium propionate solution at 0.8 g/kg BW (SP), and an infusion with sodium butyrate solution at 0.5 g/kg BW (SB) according to previous studies (Fig. 1A) (22, 44, 45). The concentration of the SCFA infusion solution was the optimal concentration screened in related studies in ruminants. The same volume of SCFAs and normal saline solutions at 1 L were slowly perfused into the rumen with a rumen tube daily before morning feeding. All solutions were calibrated to be consistent with a pH meter before infusion. The experiment lasted for 13 days, which combined with a 12-day infusion period and a 1-day sampling period. All goats received the same housed conditions except for the different SCFA-infusion treatments, and they had no history of gastrointestinal diseases or records of antibiotic usage.

### Sample collections.

All goats were fasted for 12 h before the sampling day. The animals were then weighed, slaughtered, and dressed by professional butchers as we previously described on sampling day (46). An ~100 mL of rumen contents was collected from each goat, and the samples were strained through four layers of sterile cheesecloth (47). The pH of the rumen contents was measured immediately using a calibrated pHS-3C precision pH meter (Leimeg, Shanghai, China). The strained rumen contents were then collected and stored at −80°C for the measurements of rumen fermentation parameters and the analysis of the rumen microbiome and metabolome. The entire rumen tissue was carefully separated and rinsed in phosphate-buffered saline (PBS) and subsequently arranged on an ice-cold surface. The tissue was then weighed, and the rumen index was calculated according to a previously described equation (g of rumen/kg of final BW). Next, a 2 cm × 2-cm rumen epithelial tissue samples were fixed in 4% precooled paraformaldehyde (pH 7.4) for morphology analysis. Finally, another part of 5 g of rumen epithelial tissue was rapidly frozen in liquid nitrogen for measurements of SCFA transporter genes, antioxidant capacity, inflammatory cytokines, tight-junction proteins, rumen epithelial RNA-seq, and metabolome analyses.

### Measurements of ammonia nitrogen and short-chain fatty acids of rumen contents.

The rumen content supernatants were harvested after centrifugation at 13,400 × *g* at 4°C for 10 min to determine the ammonia nitrogen and SCFA concentrations. A colorimetric method was conducted to detect the concentration ammonia nitrogen, as previously described (48). The SCFA concentration was determined according to a preliminary method of our laboratory using a gas chromatograph (GC-9A; Shimadzu, Kyoto, Japan) (49). Briefly, 1 mL of the supernatant was collected and filtered through a 0.25-μm-pore-size syringe filter. Then, 0.2 mL of metaphosphoric acid, which contained 20% of 60 mmol/L crotonic acid, was added, and the entire contents were vortexed, centrifuged, and filtered. The supernatant was then collected for subsequent analyses. Portions (0.4 μL) of test samples and standard solution mix were finally run through a CP-WAX capillary column (length, 30 m; inner diameter, 0.53 mm; film thickness, 1 mm) in a gas chromatograph. Program settings and the calculations were done according to a previously described protocol (50). The ammonia nitrogen and SCFA concentrations were calculated using the average mean at different sampling time points.

### Quantitative RT-PCR analysis of rumen epithelial tissue.

Total RNA was extracted from 50 mg of rumen epithelial tissue using a FastPure cell/tissue total RNA isolation kit V2 (RC112; Vazyme, Nanjing, China) to detect the expression level of the SCFA transporter genes. The purity and concentration of total RNA were detected using a NanoDrop spectrophotometer (Thermo Fisher Scientific, Waltham, MA). The reaction system for reverse transcription was adding 0.4 μL of RT mix, 1,000 ng of total RNA, and RNase-free ddH_2_O to make the volume of 20 μL, using a FastKing gDNA Dispelling RT Super Mix (Tiangen, Beijing, China). The reaction procedure was set as 42°C for 15 min and 95°C for 3 min.

The gDNA were used as the templates for RT-PCR by using a 2×TSINGKE Master qPCR mix (SYBR green I, TSE201; Tsingke, Beijing, China) with an ABI7500 (Thermo Fisher) sequence detector. The reaction system for the PCR was as follows: qPCR mix (10 μL); forward/reverse primer, 0.8 μL (10 μM); 50× ROX reference dye, 0.4 μL; and ddH_2_O, up to 20 μL. The PCR procedure followed a previously described protocol (43). A standard curve method and QuantStudio 7 Flex real-time PCR software (Applied Biosystems, Pleasanton, CA) were used for data analysis according to the 2^−ΔΔ^*^CT^* method (51). The specific primers for genes *SLC16A1*, *SLC16A3*, *SLC9A1*, *SLC9A3*, *SLC4A2*, *ATP6V1E1*, and *ATP1A1* are shown in Table S6 in the supplemental material.

### Morphology analysis of rumen epithelial tissue.

The procedure for hematoxylin and eosin (HE) staining in rumen epithelial tissue was based on a previous study (52). Briefly, the exemplars of the rumen epithelial were fixed via 4% paraformaldehyde solution and then embedded in paraffin, sectioned, and dyed with HE for morphology analysis using an inverted fluorescence microscope (IX71; Olympus, Tokyo, Japan). CellSens Dimension (v2.3) software was used to measure and record the data of morphometric analysis in the rumen papilla. For each sample, complete photomicrographs of rumen papilla from six visual fields were randomly measured to calculate the average mean lengths and widths of rumen papillae, SCT, SGT, SST, and SBT.

### Measurement of antioxidant capacity of rumen epithelial tissue.

The measurement of antioxidant capacity in rumen epithelial tissue was followed our previous protocol (53). Briefly, 0.1 g of rumen epithelial tissue was immediately extracted and homogenized with 1 mL of PBS buffer (0.01 mol/L; pH 7.2 to 7.4) on ice, and the homogenized tissue was then centrifuged at 13,400 × *g* at 4°C for 10 min to collect the supernatant for subsequent analysis. The protein concentration was measured using a total protein quantitative assay kit (A045-2; Nanjing Jiancheng Bioengineering Institute, Nanjing, China). The T-AOC (BC1315; Beijing Solarbio Science & Technology Co., Ltd., Beijing, China), the activity of GSH-Px (BC1195; Solarbio), and the MDA content (BC0025; Solarbio) were assayed using biochemical kits according to the manufacturers’ protocols.

### ELISA of inflammatory cytokines and tight-junction proteins of rumen epithelial tissue.

To assess the concentrations of inflammatory cytokines and tight-junction proteins, the rumen epithelial tissue was collected to implement ELISA using the supernatant after extraction and homogenizization with PBS (10%), referring to the IL-1β, IL-6, IL-10, TNF-α, Claudin1, occludin, and ZO-1 ELISA detection kit instructions (Mlbio, Shanghai, China). The immunological parameter assays were manually performed and measured the absorbance using a multifunctional microplate reader (SpectraMax M5; Molecular Devices, Sunnyvale, CA) according to a previously described protocol (54). Parameters were normalized by the total protein concentration to determine the concentrations of inflammatory cytokines and tight-junction proteins per mg of protein for each sample.

### 16S rRNA amplicon sequencing and data processing in rumen contents.

Microbial DNA of rumen contents was extracted by using a TIANamp stool DNA kit (DP328; Tiangen) according to the manufacturer’s instructions. The DNA quality was detected by agarose gel electrophoresis and quantified using a UV spectrophotometer. The 16S rDNA high-throughput sequencing was performed using an Illumina NovaSeq PE250 (Illumina, San Diego, CA) by LC-Bio Technology Co., Ltd. (Hangzhou, Zhejiang, China). The V3-V4 region of the prokaryotic small-subunit (16 S) rRNA gene was amplified by using the primers 341 F (5′-CCTACGGGNGGCWGCAG-3′) and 805 R (5′-GACTACHVGGGTATCTAATCC-3′) according to the manufacturer’s recommendations (55).

Paired-end reads generated from Illumina platforms were processed and merged using FLASH software (v1.2.7) (56). Quality filtering on the raw reads was then performed under specific filtering conditions to obtain the high-quality clean tags according to the Fqtrim software (v0.9.4). Chimeric sequences were filtered using VSEARCH software (v2.3.4) (57). Finally, the feature table and feature sequence were obtained after dereplication using DADA2 software (v1.10.1) (58). The α-diversity was used to assess the complexity of species diversity for each sample via three indices (Chao1, Shannon, and Simpson) in QIIME2 software (v2019.4), and rarefaction curves of ASV numbers and Chao1, Shannon, and Simpson indexes were generated to assess the depth of sequencing (59). The β-diversity was calculated using PCoA analysis based on the unweighted UniFrac distance (60). ASV profiling analysis and microbial relative abundance analysis were performed using R version 4.2. Finally, LEfSe analysis was used to compare the marker species under the genus classification (LDA score > 2.5) (61).

### Analysis the relative abundance of SCFA-synthetic enzymes in rumen contents.

The relative abundance of SCFA-synthetic enzymes in rumen contents was predicted and calculated based on the metabolic function profile using a metagenomic approach that named PICRUSt2 according to a previously described method (62). PICRUSt2 is a new approach for functional genes prediction for 16S rRNA which has been proven to be more accurate than any other approaches overall in metagenome inference (63). In our study, most of the SCFA synthesis-related enzymes were identified in KEGG and Metacyc databases, such as acetate (EC 6.3.4.3, EC 1.5.1.5, and EC 1.5.1.20), propionate (EC 5.4.99.25, EC 5.1.99.1, and EC 6.2.1.1), and butyrate (EC 2.3.1.9, EC 1.1.1.157, and EC 4.2.1.17). The relative abundance of SCFA-synthetic enzymes was the sum of the abundance of the genes that encode the key enzymes (62, 64).

### LC-MS untargeted metabolome analysis and data processing of rumen contents and rumen epithelial tissue.

Briefly, 100 mg of rumen contents and epithelial tissue samples were first treated with 600 μL of methanol solution. Sample preparation was carried out as we described previously (65). LC-MS untargeted metabolome analysis was performed using a Vanquish UHPLC system (Thermo Fisher Scientific, Santa Clara, CA) with an Acquity UPLC HSS T3 column (150 mm × 2.1 mm, 1.8 μm; Waters, Milford, MA) at a column temperature of 40°C, a flow rate of 0.25 mL/min, and an injection volume of 2 μL at Panomix Biomedical Tech Co., Ltd. (Suzhou, Jiangsu, China).

For LC-ESI (+)-MS analysis, the mobile phases consisted of 0.1% formic acid in acetonitrile (vol/vol) (phase C) and 0.1% formic acid in water (vol/vol) (phase D). Separation was conducted under the following gradient: 0 to 1 min, 2% C; 1 to 9 min, 2 to 50% C; 9 to 12 min, 50 to 98% C; 12 to 13.5 min, 98% C; 13.5 to 14 min, 98 to 2% C; and 14 to 20 min, 2% C. For LC-ESI (–)-MS analysis, the analytes were carried out with 5 mM acetonitrile (phase A) or 5 mM ammonium formate (phase B). Separation was conducted under the following gradient: 0 to 1 min, 2% A; 1 to 9 min, 2 to 50% A; 9 to 12 min, 50 to 98% A; 12 to 13.5 min, 98% A; 13.5 to 14 min, 98 to 2% A; and 14 to 17 min, 2% A (66).

Mass spectrometric detection of metabolites was performed on a QExactive HF-X (Thermo Fisher Scientific) with an ESI ion source. Simultaneous MS1 and MS/MS (Full MS-ddMS2 mode, data-dependent MS/MS) acquisition was used. The parameters were as follows: sheath gas pressure, 30 arbitrary (arb); aux gas flow, 10 arb; spray voltage, 3.50 kV and −2.50 kV for ESI(+) and ESI(–), respectively; capillary temperature, 325°C; MS1 range, *m/z* 81 to 1,000; MS1 resolving power, 60000 FWHM; number of data dependent scans per cycle, 8; MS/MS resolving power, 15,000 FWHM; normalized collision energy, 30%; and dynamic exclusion time, automatic (67).

The raw data were first converted to mzXML format by MSConvert in ProteoWizard software package (v3.0.8789) and processed using XCMS for feature detection, retention time correction, and alignment (68, 69). The metabolites were identified by accuracy mass (<30 ppm) and MS/MS data, which were matched with public databases, such as HMDB, massbank, LipidMaps, mzcloud, and KEGG.

For multivariate statistical analysis, normalized data were imported into soft independent modeling of class analogy (SIMCA) software (v14.1; AB Umetrics, Umea, Sweden) and preprocessed by PAR scaling and mean centering before PCA (70). OPLS-DA was also performed to allow the determination of discriminating metabolites using VIP. All the models evaluated were tested for overfitting with methods of permutation tests. Then, parametric tests were performed on normally distributed data using Welch’s *t* test, and nonparametric tests were performed on non-normally distributed data using a Wilcoxon Mann-Whitney test to calculate the statistical significance (*P*). The *P* value, VIP, and fold change (FC) were applied to discover the contributable variable for classification. Finally, metabolites with a *P* value of <0.05 and VIP values of >1 were considered to be statistically significant metabolites. The functions and metabolic pathways were enriched in KEGG database with the MetaboAnalyst 5.0 website (71). The hierarchical clustering was generated using the R package Pheatmap.

### RNA-seq and data processing in rumen epithelial tissue.

RNA-seq of rumen epithelial tissue was performed on a DNBSEQ-T7 sequencer platform (BGI, Shenzhen, China) at Novogene Co., Ltd. (Beijing, China). Briefly, total RNA for rumen epithelial transcriptome sequencing was isolated and purified according to above-described protocols. The RNA amount and purity were quantified by using a NanoDrop ND-1000 spectrophotometer. The RNA integrity was assessed by using an Agilent 2100 with an RIN number of >7.0. The cDNA library was constructed and then sequenced on the DNBSEQ-T7 platform.

The Fastp software (v0.23.1) (72) was used to perform quality control on the raw data and obtain clean data. The HISAT2 software (v2.1.0) (73) was used to align the obtained clean data to the latest reference genome (*Capra hircus* reference genome ARS1.2) (74). The SAMtools software (v1.10) was used to sort and convert the SAM files to BAM format (75). StringTie software (v2.2.1) was used to assemble and quantify the transcripts and genes based on read counts (73). Finally, the expression levels of all the mRNA and the identification of DEGs were estimated using DESeq2 package (1.36.0) in R software (v4.2). Genes that passed a threshold of *P < *0.05 and |log_2_-fold change| > 1 in DEG analysis were considered for further analysis. Finally, the functional enrichment analysis of up- and downregulated DEGs was visualized using the KEGG database.

### Statistical analysis and visualization.

All data are expressed as means ± standard deviations (SD). Significant and extremely significant differences were declared at *P < *0.05 and *P < *0.01, respectively. Comparison of the differences in rumen weight and index, rumen fermentation parameters, SCFA concentration, relative abundances of SCFA synthetic enzymes, expression of SCFA transporter genes, and rumen epithelial homeostasis parameters were subjected to the one-way analysis of variance, while microbial α-diversity indexes were conducted using Kruskal-Wallis tests with SPSS 16.0 software. Correlations between selected parameters and multi-omics data were calculated using Pearson’s or Spearman correlation coefficients. R package ggplot2, Pheatmap, ggcor, and Prism 6.0 software (GraphPad, San Diego, CA) were used for graphics.

### Data availability.

The RNA-seq data have been deposited in NCBI Gene Expression Omnibus (GEO) database under accession code GSE221507, while the 16S rRNA sequencing data have been deposited in GEO database under accession code GSE221508. The LC-MS metabolome data were deposited in the OMIX database of China National Center for Bioinformation under accession number OMIX004114. Additional data related to this paper may be requested from the authors.
