# Supplementary material for: Multi-Omics Reveals the Impact of Exogenous Short-Chain Fatty Acid Infusion on Rumen Homeostasis: Insights into Crosstalk between the Microbiome and the Epithelium in a Goat Model
Source: Microbiol Spectr. 2023 Jul 13;11(4):e05343-22. doi: 10.1128/spectrum.05343-22 (PMC10433986; doi:10.1128/spectrum.05343-22)

**Table S5: Dietary composition and nutrient levels**

| Ingredients | Percent, % | Nutrients^2^ | Level, % | |
| --- | --- | --- | --- | --- |
| Oat grass | 41.00 | DE/(MJ/kg) | 11.01 |  |
| Corn | 29.5 | CP | 36.00 |  |
| Soybean meal | 14.5 | NDF | 20.5 |  |
| Wheat bran | 4.00 | ADF | 29.48 |  |
| Stone dust | 0.35 | EE | 2.22 |  |
| CaH_2_PO4 | 0.15 | Ca | 0.44 |  |
| NaCl | 0.50 | P | 0.35 |  |
| Premix^1^ | 4.00 |  |  |  |
| Total | 100.00 |  |  |  |

^1^ The premix was provided following per kg of diet: VA 200,000 IU, VD3 70,000 IU, VE 350 IU, Fe 1.6 g, Cu 1.7 g, Zn 8.2 g, Mn 2.5 g, and Se 40 mg.

^2^ DE in diet was calculated according to ingredient composition, while nutrient levels of the other nutrients were measured values.

**Table S6: Specific primers used for RT-PCR**

| Genes | 5’ - 3’ | Products length |
| --- | --- | --- |
| *SLC16A1* | F: ACCAGTTTTAGGTCGTCTCA  R: GGCTTCTCAGCAACATCTACA | 207 |
| *SLC16A3* | F: CTACAGAGATGTCTTCAAGGGTT  R: GCTAAATACGAGCGTTGACGG | 118 |
| *SLC9A1* | F: CGGGCATCATGGCACTCATT  R: TGATGGTCGTGTGGGACTTG | 87 |
| *SLC9A3* | F: CTGCAGACTTGACGTCGCC  R: TAGGAGGGTGGCCGTGATAG | 210 |
| *SLC4A2* | F: AGCAGCAACAACCTGGAGT  R: GGTGAAACGGGAGACGAA | 123 |
| *ATP1A1* | F: CCTCGAAATCCATTGCTTATACC  R: GACCATGTTCCGTTCCCAAGT | 137 |
| *ATP6V1E1* | F: TTTTATTGAACAAGAAGCCAATGA  R: GATTCATCAAATTGGACATCTGAA | 182 |
| *GAPDH* | F: CAAAGTGGACATCGTTGCCA  R: TGGAAGATGGTGATGGCCTT | 156 |

**Figure S1: The light micrographs and structure of rumen papillae cross-sections**


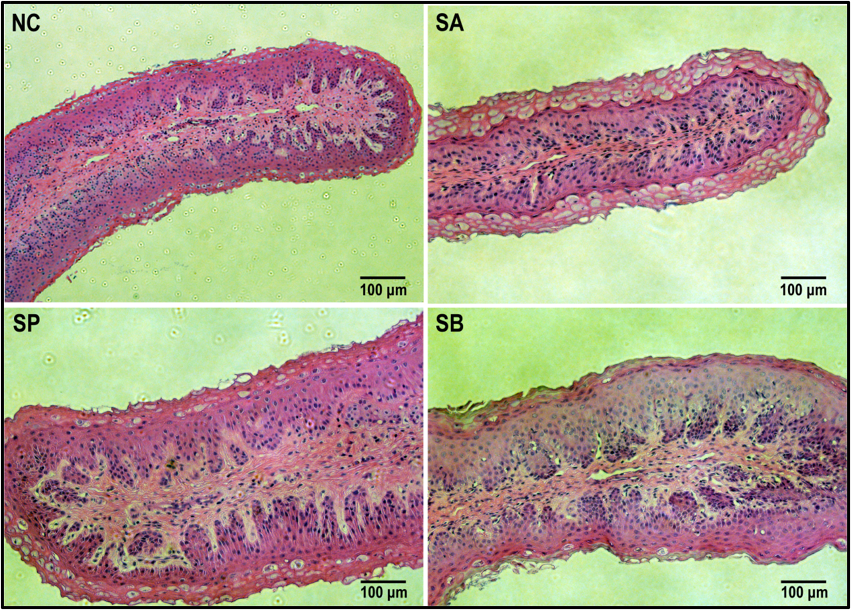


**Figure S2: The standard curves for the enzyme activities of total proteins, tight junctions, and inflammatory cytokines in rumen epithelial tissue measured by ELISA method**


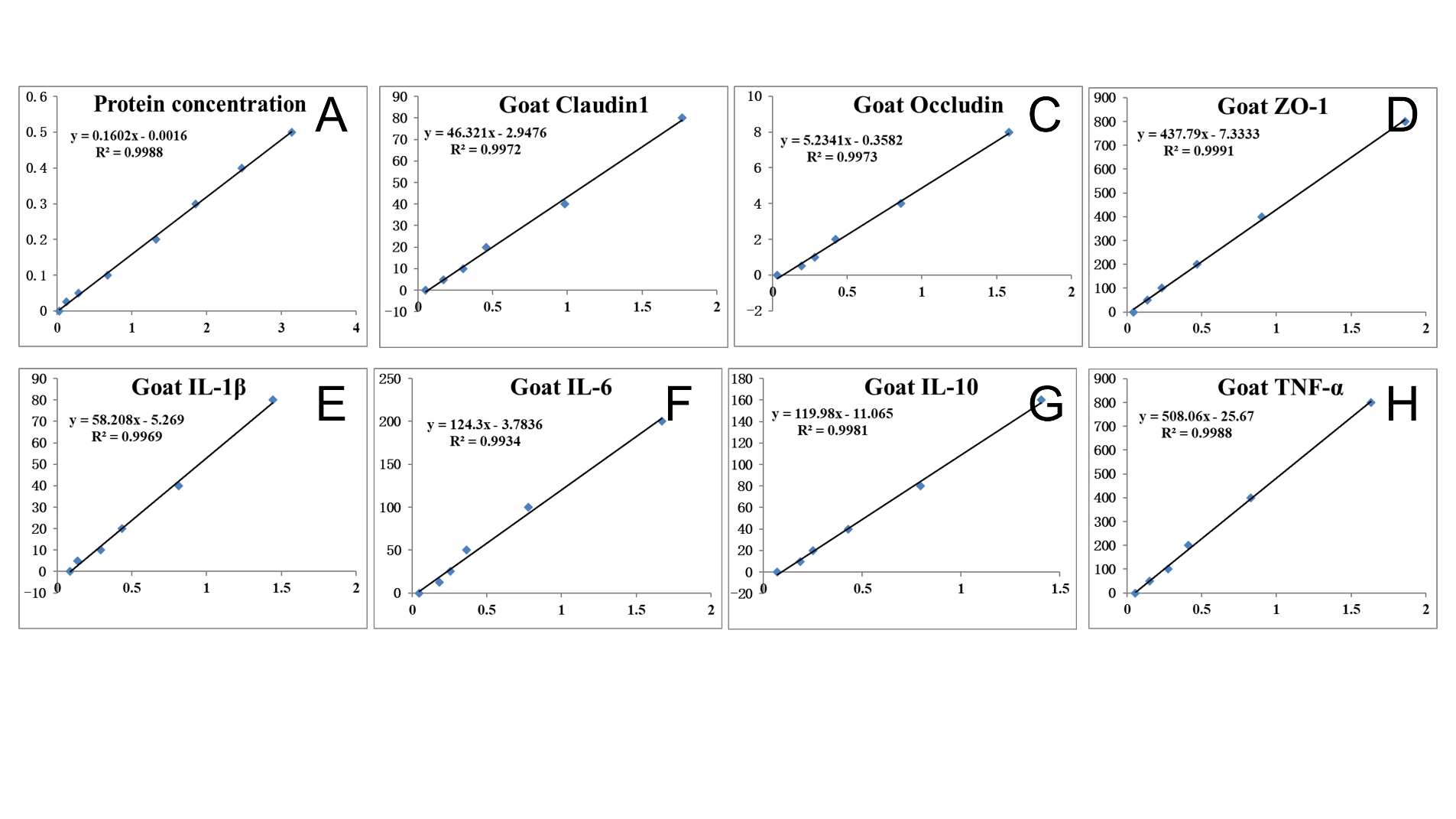


**Figure S3:** **The observed ASVs, sequencing coverage, rarefaction curve, and the relative abundance of Bacteroidetes and Firmicutes in rumen contents**


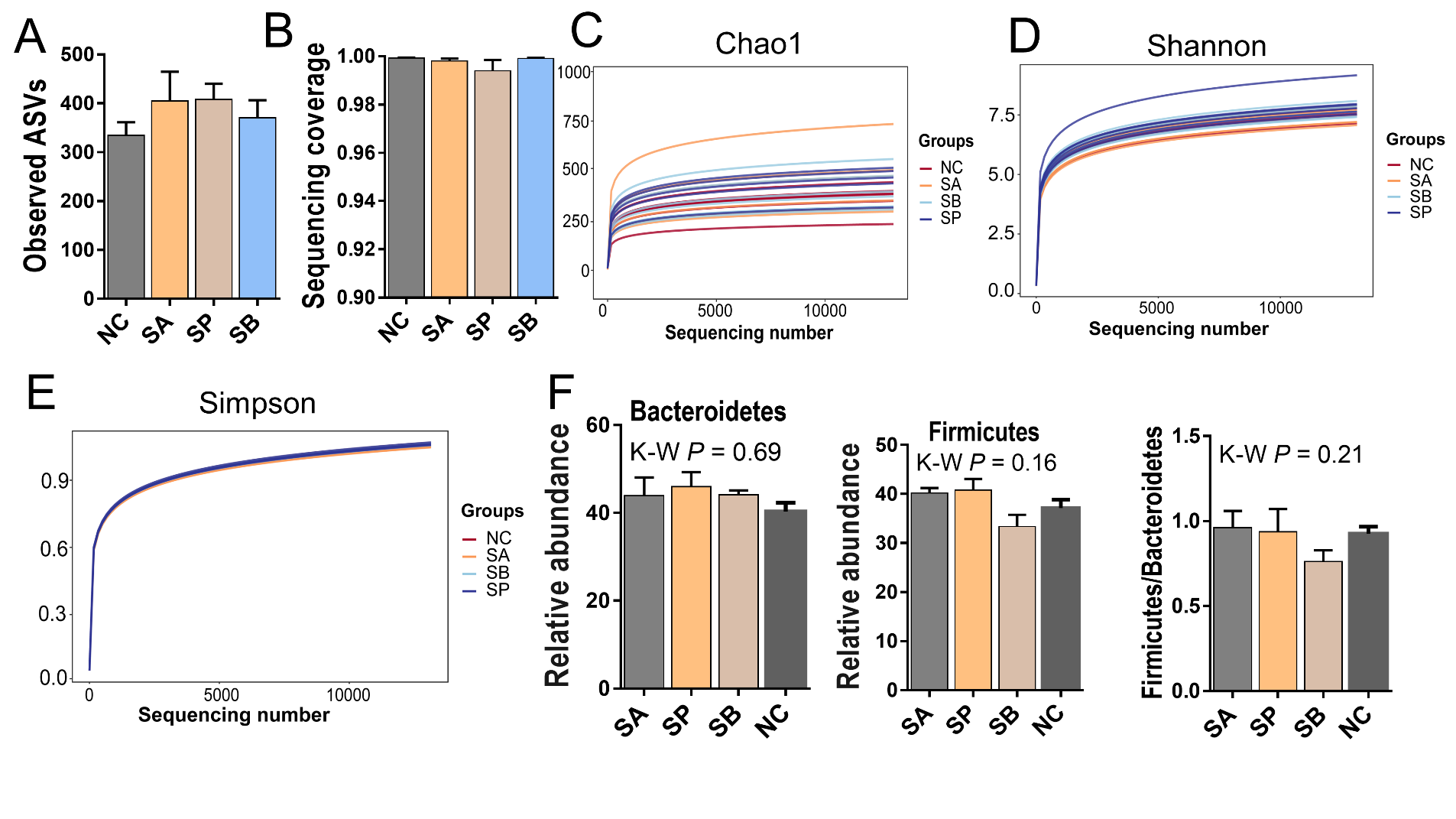


**Figure S4: The distributions of microbiome data using PCA and the permutation test of OPLS-DA score plot among four groups in rumen contents**


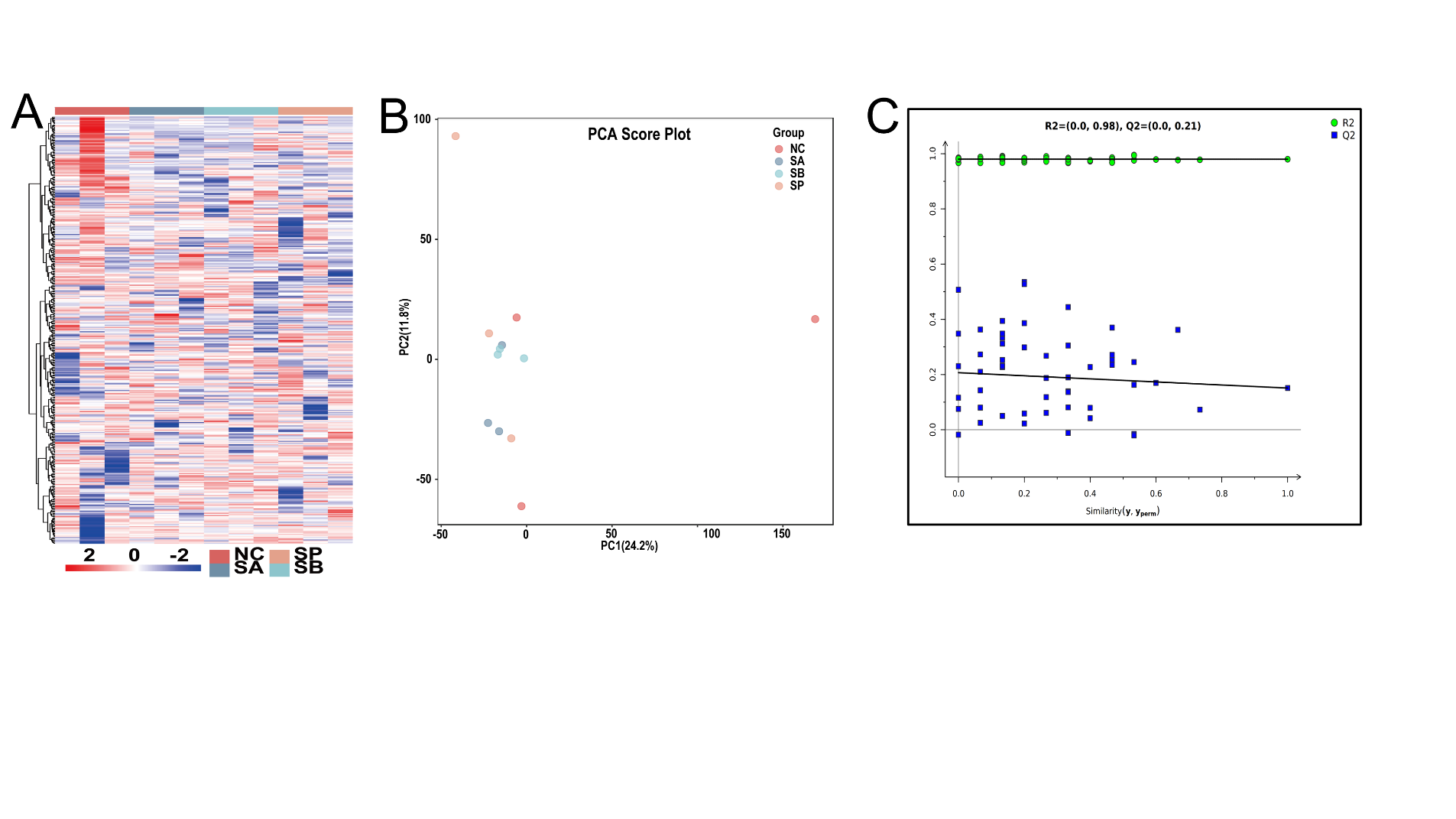


**Figure S5: Heatmap of correlation analysis between the differential metabolites and rumen bacteria**


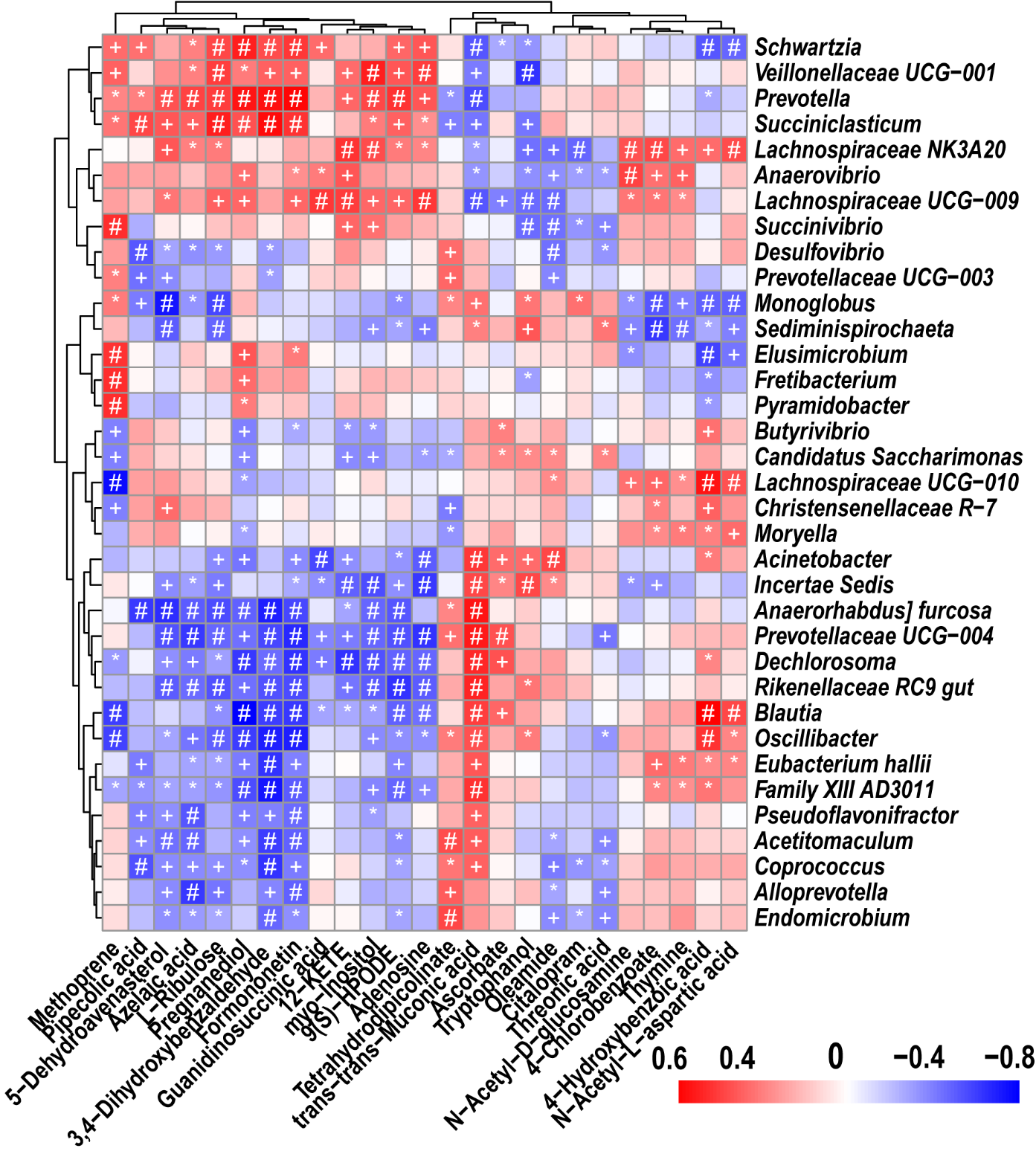


**Figure S6: The total observed metabolites, distributions of microbiome data using PCA and OPLS-DA score plot, and the permutation test of among four groups in rumen epithelium**


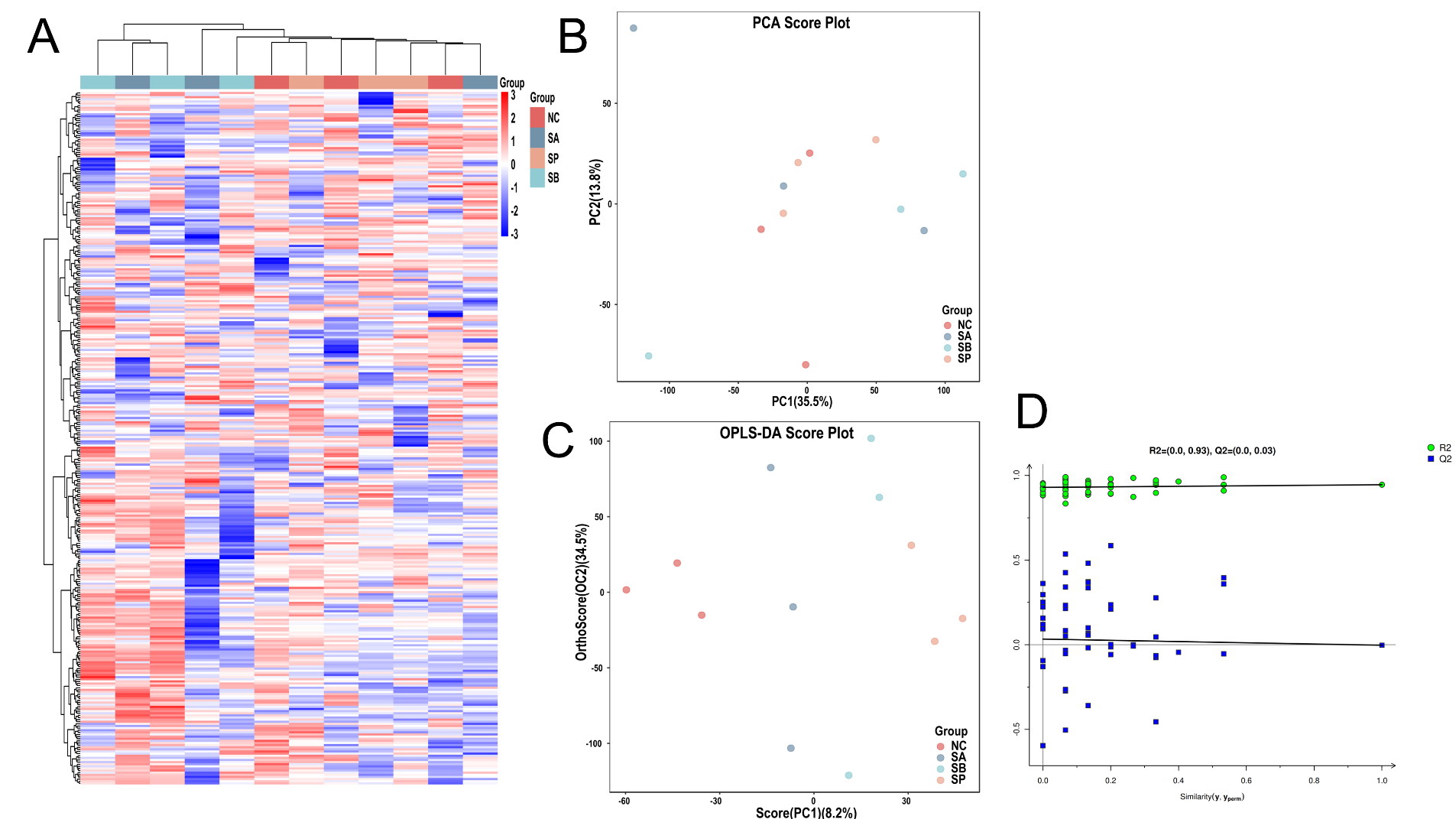

Supplement: Supplemental file 5 — Supplemental material. Download spectrum.05343-22-s0005.docx, DOCX file, 4.0 MB [file spectrum.05343-22-s0005.docx]
